# Digital phenotyping of CGM engagement reveals distinct glycemic outcomes

**DOI:** 10.1371/journal.pdig.0001505

**Published:** 2026-07-23

**Authors:** Bowen Zhang, Tomoki Okuno, Emma Li, Ethan Riggins, Evie L. Shen, Joleen Vansomphone, Estelle M. Everett, Gregory J. Norman, Donald R. Miller, Peter D. Reaven, Hua Zhou, Jin J. Zhou

**Affiliations:** 1 Department of Biostatistics, University of California, Los Angeles, California, United States of America; 2 Phoenix VA Health Care System (111E), Phoenix, Arizona, United States of America; 3 Castilleja School, Palo Alto, California, United States of America; 4 Department of Mathematics, University of California, Berkeley, California, United States of America; 5 Union County Magnet High School, Scotch Plains, New Jersey, United States of America; 6 Huntington Beach High School, Huntington Beach, California, United States of America; 7 David Geffen School of Medicine, University of California, Los Angeles, California, United States of America; 8 VA Greater Los Angeles Health Care, Los Angeles, California, United States of America; 9 Dexcom, Inc., San Diego, California, United States of America; 10 Department of Biomedical and Nutritional Sciences, University of Massachusetts, Lowell, Massachusetts, United States of America; 11 VA Center for Medication Safety, Department of Veterans Affairs, Chicago, Illinois, United States of America; University of Exeter, UNITED KINGDOM OF GREAT BRITAIN AND NORTHERN IRELAND

## Abstract

Benefits from continuous glucose monitors (CGMs) may depend on how devices are used over time—not only how often they are used. We linked one year of device-generated CGM wear data from 2,351 U.S. Veterans to electronic health records (EHRs) to characterize real-world usage during the first year after CGM initiation. To compare longitudinal use patterns in the presence of unsynchronized sensor replacement–related gaps and intermittent interruptions, we aligned daily wear streams using a complexity-adjusted, time-adaptive optimal transport (TAOT) distance and applied spectral clustering to identify data-driven usage phenotypes. To estimate the adjusted association between CGM usage phenotypes and clinical outcomes, we used a double/debiased machine learning framework to quantify 12-month changes in time in range (ΔTIR) and mean glucose (ΔMG). We identified three reproducible patterns of CGM usage: Consistent, Fluctuating, and Low engagement. Relative to Consistent wear, Fluctuating usage was associated with worse glycemic change (ΔTIR = −3.56%, 95% CI: −4.76 to −2.36; ΔMG = +7.12 mg/dL, 95% CI: 4.91 to 9.32), and Low engagement showed larger deterioration (ΔTIR = −7.00%, 95% CI: −10.80 to −3.14; ΔMG = +14.09 mg/dL, 95% CI: 6.52 to 21.67). Notably, 73.2% of Fluctuating users still met a common “adherence” threshold (≥80% days worn), indicating that simple coverage metrics can miss clinically relevant instability. The differences in glycemic change (ΔTIR and ΔMG) between the Consistent and Fluctuating groups were most pronounced among subgroups with more intensive diabetes management needs, such as insulin pump or glucagon users, suggesting that sustained CGM usage may be particularly important when clinical management is more complex. Beyond CGM and diabetes, this work provides a generalizable framework for characterizing longitudinal usage patterns of intermittently used digital health technologies and linking derived usage phenotypes to clinical outcomes. The approach can support more precise evaluation, monitoring, and intervention design for a wide range of real-world digital health tools.

## Author summary

Continuous glucose monitors, or CGM, are widely used in diabetes care, yet evidence linking long-term CGM usage pattern to glycemic control remains limited. In this study, we examined long-term real-world data from more than 2,300 U.S. Veterans who used CGM for up to one year. Rather than summarizing use with a single adherence percentage, we looked at day-to-day patterns of use and how stable or irregular those patterns were over time. We identified three distinct groups: individuals with consistent use, a fluctuating group characterized by irregular engagement with interruptions or insufficient daily wear, and a low-engagement group. Importantly, many people in the fluctuating group would still be considered “adherent” by standard definitions, while they experienced worse glucose outcomes than consistent users, highlighting the limitations of common adherence thresholds. Consistent use was especially beneficial for individuals with more complex diabetes management needs, suggesting that targeted support to sustain stable use may yield greater clinical benefit. Overall, our work highlights the importance of understanding patterns of usage with wearable devices and introduces a general, data-driven framework for linking real-world usage trajectories to clinical outcomes.

## Introduction

Wearable health technologies offer unprecedented opportunities for real-time monitoring of chronic conditions. In diabetes care, continuous glucose monitoring (CGM) systems have demonstrated clinical efficacy in improving glycemic control, reducing hypoglycemia, lowering hospitalization rates, and reducing diabetes complications [1–5]. However, these benefits are contingent on sustained engagement, often on a near-daily basis, for more than 70% of the time [1,2,6–12] (See S1 Table). Real-world adherence, however, often falls short of clinical trial standards.

In practice, maintaining such high levels of adherence has proven challenging, and real-world usage patterns are less uniform and frequently decline over time [13]. For instance, a 6-month study of 38 individuals with type 1 diabetes on sensor-augmented pump therapy reported that CGM adherence is highly individualized and tends to drop around 9–11 weeks after initiation [14]. Similarly, in another study, participants were considered adherent if they wore the sensor for at least 60% of the time, yet only 61% achieved this threshold over one year [15]. More recently, a retrospective analysis of 559 individuals with type 1 or type 2 diabetes found that only 51% were adherent users (defined as sensor wear on >80% of days) during the first year of CGM use [16].

Unlike medications, which are guided by clear dosing schedules, wearable devices lack a standardized way to define or measure use. Patterns of usage vary widely across individuals, and current adherence metrics, such as the number of days the device is worn, often rely on arbitrary thresholds. These measures may not capture whether the device was used in a way that supports clinical effectiveness. As a result, it is difficult to distinguish consistently engaged users from those with sporadic use that may limit clinical benefit. Without reliable measures of adherence, both the clinical impact of wearables and the value of the data they produce remain uncertain.

Real-world evidence on long-term CGM use remains limited, as most observational studies span only days to weeks, which is insufficient to capture how adherence patterns evolve and impact outcomes over time. Moreover, existing studies often adjust for only a limited set of covariates, limiting their ability to account for confounders that influence both CGM use and glycemic control. To address this gap, we analyzed one of the largest CGM usage cohorts to date: more than 2,300 patients from the U.S. Department of Veterans Affairs (VA), with a median follow-up of three years [17,18]. This longitudinal dataset allowed for a granular characterization of wear behaviors beyond simple aggregate metrics, and was complemented by detailed electronic health record (EHR)-based covariates that enabled adjustment for potential confounders.

We developed a data-driven framework to characterize long-term CGM wear patterns and assess their effect on glycemic control. Rather than relying on cumulative adherence metrics, our approach models the full temporal structure of daily CGM use to identify distinct usage phenotypes and estimate their associations with one-year changes in CGM-derived glycemic outcomes, adjusting for potential confounders.

Although demonstrated in the context of CGM use for diabetes, the proposed framework is broadly applicable to other intermittently worn biosensors. By capturing both overall usage and day-to-day variability in device engagement, it provides a general approach for analyzing real-world wearable data in settings where adherence is heterogeneous and dynamically evolving.

## Methods

To extract meaningful insights from these data, we designed a fully data-driven analytical pipeline integrating trajectory similarity modeling, clustering, and outcome modeling. Pairwise dissimilarity between individual CGM wear was quantified using a complexity-adjusted time-adaptive optimal transport (TAOT) distance, which served as input to spectral clustering for identification of distinct usage phenotypes. A double/debiased machine learning (DML) framework was subsequently used to estimate the adjusted association of each usage phenotype with one-year changes in CGM-derived glycemic outcomes.

### Ethics statement

This study was reviewed and approved by the Research & Development Committee (R&DC) of the Phoenix VA Health Care System, Department of Veterans Affairs. Initial approval was granted on December 14, 2021, and the study procedures reported here were approved through a subsequent protocol amendment on August 14, 2025.

The study involved human participants through secondary analysis of data collected under an approved VA research protocol. A waiver of informed consent and HIPAA authorization was granted by the Phoenix VA Health Care System R&DC because the study posed minimal risk to participants and used deidentified data maintained behind VA firewalls. No verbal or written consent was required or obtained.

### Data source and study design

We conducted a retrospective observational study linking CGM data with EHR from the U.S. VA Healthcare System. CGM data obtained from Dexcom, Inc. spanned October 2015–April 2023 and linkage with EHR data was performed using crosswalk identifiers to harmonize datasets at the individual level [17].

EHR data were obtained from the VA Corporate Data Warehouse (CDW), a national repository containing detailed clinical and administrative information from inpatient and outpatient encounters, including diagnoses, medications, laboratory results, vital signs, and demographics. The study protocol was approved by the Phoenix VA Health Care System Institutional Review Board (IRB) with a waiver of informed consent for secondary data analysis. Dexcom’s data-sharing protocol received an exempt determination from the WCG IRB.

To characterize longitudinal CGM engagement (or use patterns), we analyzed the first year after CGM initiation, defined as the date of the first device upload to the Dexcom server (Day 0). Patterns of CGM wear trajectories were identified over Days 1–365. We excluded individuals with: (i) no confirmed diagnosis of type 1 or type 2 diabetes; (ii) use of multiple CGM devices during the observation window based on EHR prescription data; (iii) insufficient wear (<6 days) in the first two weeks after CGM initiation; (iv) inadequate annual wear (<60 days); or (v) failure to link CGM and EHR records.

To assess the association between usage phenotypes and glycemic impact, we compared CGM-derived glycemic metrics between a baseline window (Days 1–14) and a follow-up window (Days 366–395).

**Fig 1:**
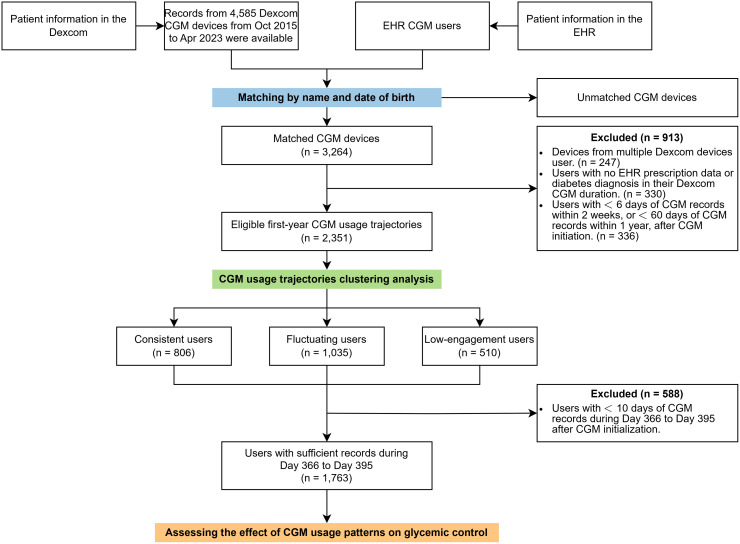
Flowchart of cohort selection. Dexcom CGM data were matched with EHR records, followed by exclusions based on multiple device use, missing EHR linkage, diabetes diagnosis, and insufficient CGM data. Eligible users underwent clustering analysis to identify distinct CGM usage trajectories, resulting in three groups: Consistent, Fluctuating, and Low-engagement users. Users with limited CGM readings during the follow-up window were excluded only from the glycemic outcome modeling due to incomplete outcome data, but were retained in the clustering stage. Abbreviations: T1D, type 1 diabetes; T2D, type 2 diabetes.

Usage phenotypes were identified using first-year CGM trajectories, where all included participants had sufficient data. Participants with fewer than 10 CGM days in the follow-up window were subsequently excluded from the glycemic outcome modeling due to insufficient data to reliably construct outcome measures, but were retained in the clustering stage. The cohort selection process is summarized in Fig 1.

### CGM-derived glycemic metrics and outcome definitions

Dexcom CGM devices measure interstitial glucose concentrations and report estimated blood glucose values at 5-minute intervals, yielding up to 288 readings per day. Glycemic outcomes were computed as changes between baseline and follow-up windows. Specifically, we assessed four CGM-derived metrics: mean glucose (MG, mg/dL), time-in-range (TIR, 70–180 mg/dL), time-below-range (TBR, < 70 mg/dL), and coefficient of variation (CV) of daily glucose levels (Table 1). Detailed equations and calculation windows were provided in S2 Table.

**Table 1:** Definition of primary outcomes.

Outcome	Definition
Change in MG	Difference in the mean glucose (mean CGM readings) between Day 366–395 and Day 1–14 following CGM initiation.
Change in TIR	Difference in the percentage of time spent in the target glucose range (70–180 mg/dL) between Day 366–395 and Day 1–14.
Change in TBR	Difference in the percentage of time spent below the glucose threshold (<70 mg/dL) between Day 366–395 and Day 1–14.
Change in CV	Difference in the weighted average daily CV between Day 366–395 and Day 1–14, with weights proportional to daily CGM record counts.

All glycemic metrics were derived from CGM data and computed as changes between baseline and follow-up windows relative to CGM initiation.

### Trajectory-based clustering of device usage

The number of CGM readings collected over time captures the fine-grained temporal structure of device engagement. To identify adherence phenotypes, we clustered individuals based on their daily CGM reading counts during the first year following initiation. A critical step in this process is selecting a distance measure which quantifies similarity between longitudinal trajectories by comparing adjacent observations over time. For CGM data, this choice must further account for the specific temporal characteristics and variability inherent to device usage. CGM sensors are replaced every 10 days, introducing regular gaps of nearly two hours due to sensor warm-up. These replacement cycles are unsynchronized across individuals, leading to temporal misalignment. Additionally, users may occasionally discontinue sensor use, resulting in sporadic interruptions in the data stream. These patterns require a distance metric capable of handling both local temporal scaling and irregular missingness. Although Dynamic Time Warping is commonly used to align unevenly sampled time series, it often produces pathological alignments [19] and, in practice, offers no clear advantage over simpler metrics like Euclidean distance for clustering purposes [20]. To address these limitations, we developed Complexity-adjusted TAOT, a method grounded in optimal transport theory that enables many-to-many temporal alignments. TAOT naturally accommodates the distortions and irregularities characteristic of CGM trajectories, providing a more robust foundation for clustering adherence patterns [21].

To further refine our distance metric, we incorporated a complexity-informed adjustment that increases sensitivity to meaningful behavioral differences in CGM usage. While temporal alignment accounts for timing discrepancies, it may overlook structural irregularities, such as frequent on-off transitions. Inspired by prior work demonstrating the benefits of complexity-aware metrics in distinguishing time series with differing structural patterns [22], our approach enhances separation between users whose CGM traces may appear superficially similar but have subtle differences related to nuances in their behavior with CGMs. While simpler interruption summaries, such as the number or duration of stoppages, are clinically interpretable, they capture only pre-specified aspects of device nonuse and may miss broader temporal instability, including fragmented wear, repeated high–low transitions, and irregularity across weekly routines or sensor-replacement cycles.

To capture structural irregularities beyond temporal alignment, we augment the TAOT distance with a measure of local complexity. As a foundation, we use Refined Composite Multiscale Entropy (RCMSE) [23], a robust entropy-based statistic that quantifies the intricacy of a time series and remains stable even for short segments, and has been applied to measure the complexity of glucose time series [24,25]. By tuning its scale and threshold parameters to reflect domain-specific rhythms—such as weekly behavioral cycles and the 10-day CGM sensor replacement interval—we obtain a context-aware complexity score that highlights clinically meaningful engagement patterns. Details on the selection of scale and threshold parameters are provided in the S1 Appendix Note 2. We then defined the complexity-adjusted TAOT distance between two trajectories as


$$\begin{array}{c} d({𝐮}_{i},{𝐮}_{j})=d_{TAOT}({𝐮}_{i},{𝐮}_{j})\cdot \left(1+\log_{b}\frac{\max\left\{RCMSE({𝐮}_{i}),RCMSE({𝐮}_{j})\right\}}{\min\left\{RCMSE({𝐮}_{i}),RCMSE({𝐮}_{j})\right\}}\right), \end{array}$$

where ${𝐮}_{i},{𝐮}_{j}\in {\mathbb{R}}^{T}$ denote CGM usage trajectories over *T* days, $d_{TAOT}(\cdot ,\cdot )$ is the TAOT distance, and RCMSE(·) is the trajectory’s complexity score. The multiplicative correction inflates distances when two trajectories differ substantially in complexity, thereby exposing behavioral differences that are not apparent from alignment alone. To calibrate the strength of this correction, we define the logarithm base *b* relative to the observed ranges of complexity and TAOT distances across the study cohort. Let U represent the set of all trajectories under study, $R_{\text{max}}=\max_{u\in U},RCMSE(𝐮)$, $R_{\text{min}}=\min_{u\in U},RCMSE(𝐮)$, $D_{\text{max}}=\max_{u,v\in U},d_{TAOT}(𝐮,𝐯)$, and $D_{\text{min}}=\min_{u,v\in U},d_{TAOT}(𝐮,𝐯)$. We then set


$$\begin{array}{c} b={\left(\frac{R_{\text{max}}}{R_{\text{min}}}\right)}^{\frac{1}{\frac{D_{\text{max}}}{D_{\text{min}}}-1}}, \end{array}$$

ensuring that the scale of the correction is adapted to the observed dynamic range in both alignment-based and complexity-based dissimilarities. As illustrated in S1 Fig, incorporating this complexity-aware adjustment allows our method to better differentiate usage patterns that are behaviorally distinct yet temporally aligned.

To identify adherence phenotypes, we then constructed a locally adaptive affinity matrix using a Gaussian kernel with bandwidths tuned to local distance structure. Clustering was performed using normalized spectral clustering [26,27].

We used a stability-based model selection approach to determine the number of clusters [28]. Spectral clustering was repeated over 50 random splits. For each split, the data were divided 1:1 into training and test sets. Clusters were estimated on the training set, used to predict test-set assignments, and then re-estimated directly on the test set. Stability was defined as the disagreement between the two test-set labelings. The optimal number of clusters was chosen to minimize the mean disagreement across splits. In addition, we evaluated cluster separation using the same pairwise distance matrix employed for spectral clustering. Specifically, we computed the mean within-cluster and between-cluster distances and summarized their ratio as a measure of separation. This metric was used as a complementary diagnostic to compare within-cluster and between-cluster variability.

For comparison with conventional adherence metrics, we summarized usage using three aggregate measures: proportion of days covered (PDC) over one year, proportion of time covered (PTC) over one year, and PTC restricted to active wear days. These provided a reference framework for evaluating our trajectory-based characterization of device engagement.

The code for usage trajectory clustering (complexity-adjusted TAOT distance and spectral clustering pipeline) is available on GitHub at: https://github.com/BowenZhang2001/CGM_usage_clustering.

### Glycemic effect estimation via a double machine learning framework

To evaluate the association between different first-year CGM usage patterns and glycemic control at 12 months, we applied a DML framework [29,30]. DML is a modern framework for estimating causal parameters in observational studies under standard identification assumptions, particularly in high-dimensional settings. It combines the flexibility of machine learning with the rigor of statistical theory to produce valid estimates, even when the relationships between variables are complex and nonlinear.

Let *k* be a prespecified fixed number of clusters, and let $D\in \{d_{1},\ldots ,d_{k}\}$ denote the CGM usage pattern cluster to which each participant belongs, with $A_{j}=1\{D=d_{j}\}$ as the indicator for assignment to usage pattern $d_{j}$. Let *X* represent a high-dimensional set of baseline covariates, including demographics, clinical characteristics, healthcare utilization, and medication use. Clinical and medication information were assessed during the year preceding initiation (24 months for the Frailty Index and Diabetes Complication Severity Index). For HbA1c, baseline was defined as the most recent measurement before CGM initiation. When unavailable, we used the closest measurement obtained within three months post-initiation. In addition, CGM-derived metrics such as MG, TIR, TBR, and CV were calculated using data from the first two weeks following initiation. Let *Y* denote the primary outcome: the 12-month change in glycemic control, defined in Table 1.

Our goal was to estimate the average potential outcome (APO) for each usage pattern $d_{j}$, defined as $\theta_{j}=𝔼[Y(d_{j})]$, where $Y(d_{j})$ represents the potential outcome that would have been observed if all participants had followed usage pattern $d_{j}$. Identification of $\theta_{j}$ requires the standard assumptions of consistency, conditional exchangeability ($Y(d_{j})⟂D|X$), and overlap ($0<\mathrm{Pr}(D=d_{j}|X)<1$) [31].

DML estimates $\theta_{j}$ by first modeling two key components using machine learning: the outcome model $g(d_{j},X)=𝔼[Y|D=d_{j},X]$, which predicts glycemic outcomes from covariates and CGM usage pattern; and the treatment (or propensity) model $m_{j}(X)=\mathrm{Pr}(D=d_{j}|X)$, which predicts the probability of receiving each usage pattern given the covariates. These models are referred to as nuisance functions because they are not of direct interest but are necessary to adjust for confounding. The DML estimator combines these predictions in an orthogonalized score function and incorporates cross-fitting, where the sample is repeatedly partitioned into folds, nuisance functions are re-estimated across folds. This procedure reduces overfitting bias and ensures valid inference even when using flexible, nonparametric learners.

Given that some participants did not have sufficient CGM readings during the 12-month follow-up window to construct outcomes, we assumed missingness at random and applied inverse-probability weighting (IPW) to adjust for informative loss to follow-up. The probability of outcome observation R=1{Adequate readings during the follow-up window} was estimated via L1-penalized logistic regression on (*D*, *X*), yielding $\hat{\pi }(D,X)$. For each participant with observed outcome ($R_{i}=1$), the normalized IP weight was computed as:


$$\begin{array}{c} w_{i}=\frac{1/\hat{\pi }(D_{i},X_{i})}{\sum_{j:R_{j}=1}1/\hat{\pi }(D_{j},X_{j})}. \end{array}$$

To reduce weight variability and improve covariates balance, we regressed $w_{i}$ on $X_{i}$ among users with observed outcome and used the fitted values as stabilized weights ${\bar{w}}_{i}=𝔼[w_{i}|X_{i}]$.

These weights were used in the DML APO estimator:


$$\begin{array}{c} {\hat{\theta }}_{j}=\frac{1}{n}\sum_{i=1}^{n}\left[w_{i}\hat{g}(d_{j},X_{i})+{\bar{w}}_{i}\frac{1\{D_{i}=d_{j}\}}{{\hat{m}}_{j}(X_{i})}\left(Y_{i}-\hat{g}(d_{j},X_{i})\right)\right]. \end{array}$$

This estimator preserves the APO estimand while correcting for selection bias due to missing outcomes and remains consistent even when either $\hat{g}$ or ${\hat{m}}_{j}$ is misspecified, a property known as double robustness.

To fit the nuisance models *g* and $m_{j}$, we compared four candidate algorithms: elastic net, random forest, support vector machine, and multilayer perceptron. These algorithms were selected as representative learners with different modeling properties. Models were evaluated using repeated 5-fold cross-validation, and final learners were selected based on their out-of-fold prediction loss and the stability of resulting APO estimates ${\hat{\theta }}_{j}$. Missingness in covariates was generally low; for variables with less than 5% missingness, imputation was done using median (numeric) or mode (categorical) values. Variables exceeding this threshold were also assigned a missing-indicator flag.

To assess effect heterogeneity, we computed group-average potential outcomes (GAPOs) within subgroups defined by diabetes type (T1D vs. T2D), age group (≥ 65 vs. < 65), insulin pump use, and glucagon prescription, as these factors capture clinically meaningful differences in disease phenotype, treatment intensity, and hypoglycemia risk that may modify how CGM engagement influences glycemic outcomes. All DML analyses were implemented in Python using the DoubleML package, with underlying learners from scikit-learn.

## Results

### Baseline characteristics of the study cohort

After applying exclusion criteria, 2,351 individuals remained for clustering analysis, while 1,763 individuals remained for glycemic effect estimation. During the first year of CGM use, only 55.8% of users had a PTC ≥80% (S2 Fig). Table 2 summarizes the baseline characteristics of the study cohort for usage trajectories clustering analysis. The majority of participants had T2D (64.8%), with a mean age of 60.7 years (SD 10.8), and 90.6% were male. The dataset captured a broad range of covariates, including race/ethnicity, geographic region, comorbidities, medication use, lab values, CGM-derived metrics, and markers of healthcare engagement such as visit frequency and no-show rates.

**Table 2:** Baseline characteristics by usage pattern (n = 2,351).

Baseline characteristics	Total	Consistent	Fluctuating	Low-engagement	P
Diabetes type (%, Type 2 diabetes)	64.8	62.4	66.3	65.5	0.210
Index Year (%)					<0.001
2015	0.3	0.1	0.5	0.0	
2016	1.0	0.9	1.4	0.2	
2017	1.2	1.0	1.3	1.6	
2018	14.8	12.4	16.9	14.3	
2019	35.9	36.2	37.7	31.6	
2020	34.4	37.2	32.9	32.7	
2021	9.3	9.8	7.6	11.8	
2022	3.2	2.4	1.6	7.8	
Age at index (years)*	60.7 (10.8)	61.7 (10.6)	60.1 (10.9)	60.0 (10.7)	0.002
Gender (% Male)	90.6	91.9	89.6	90.6	0.224
BMI (kg/m^2^)*	31.0 (6.7)	31.3 (6.3)	30.8 (6.8)	30.9 (7.0)	0.264
Ethnicity (%)					<0.001
White, Non-Hispanic	76.5	83.5	72.3	74.0	
African American	17.4	11.7	21.4	18.3	
White, Hispanic	3.9	3.1	3.9	5.1	
Other	2.2	1.7	2.4	2.6	
Region (%)					0.168
South	39.0	37.5	41.1	37.1	
Midwest	23.4	23.3	21.6	26.9	
West	22.7	24.2	22.8	20.0	
Northeast	15.0	15.0	14.5	16.1	
Endocrinologist visit (%)	85.6	85.6	86.9	83.1	0.146
Total PCP and endocrine visits^$^	6.0 [4.0, 9.0]	6.0 [4.0, 9.0]	7.0 [4.0, 10.0]	7.0 [4.0, 9.0]	0.015
LDL cholesterol (mg/dL)*	83.4 (33.2)	82.0 (31.9)	82.8 (33.1)	86.9 (35.5)	0.047
HDL cholesterol (mg/dL)*	48.4 (16.5)	48.7 (16.4)	48.1 (16.5)	48.3 (16.5)	0.806
Total cholesterol (mg/dL)*	156.7 (41.6)	154.3 (39.6)	156.8 (41.9)	160.5 (43.7)	0.052
Triglycerides (mg/dL)^$^	111.0 [75.0, 176.0]	105.0 [72.0, 166.0]	114.0 [74.0, 180.0]	118.0 [81.0, 185.5]	0.015
SBP (mmHg)*	134.0 (13.7)	133.7 (13.7)	134.2 (13.5)	133.8 (13.9)	0.712
DBP (mmHg)*	75.3 (7.8)	75.0 (7.6)	75.4 (8.0)	75.5 (7.6)	0.386
Creatinine (mg/dL)^$^	1.1 [0.9, 1.4]	1.1 [0.9, 1.3]	1.1 [0.9, 1.4]	1.1 [0.9, 1.4]	0.986
HbA1c (%)*	8.4 (1.6)	8.0 (1.3)	8.6 (1.7)	8.5 (1.7)	<0.001
Any insulin use (%)	91.8	93.2	92.3	88.8	0.015
Insulin pump (%)	23.2	25.8	21.5	22.5	0.092
Dual basal and bolus use (%)	60.0	60.0	61.6	56.7	0.172
Long/basal insulin (%)	59.7	59.2	62.1	55.7	0.049
NPH insulin (%)	3.2	3.8	2.2	4.1	0.058
Mixed insulin (%)	2.6	2.2	2.4	3.5	0.315
Short/rapid insulin (%)	84.5	86.0	85.9	79.2	0.001
Basal use only (%)	3.4	3.3	3.2	3.9	0.752
Glucagon use (%)	23.4	22.0	23.3	25.9	0.260
Statin use (%)	77.0	76.1	77.1	78.2	0.653
Anti-hypertensive medication use (%)	75.3	76.3	75.6	73.1	0.416
Noninsulin DM medication use (%)	43.4	42.3	44.3	43.1	0.676
Hypoglycemic risk score – Low (%)	79.0	81.6	77.4	78.2	0.075
Private insurance (%)	46.9	43.8	49.0	47.6	0.081
Medicare (%)	49.2	50.0	49.0	48.4	0.841
Medicaid (%)	1.3	1.2	1.4	1.0	0.738
Extended Care (%)	8.6	5.7	9.4	11.8	<0.001
Smoking status (%)					0.007
Never smoker	33.7	30.4	35.5	35.3	
Former smoker	48.1	53.8	44.4	46.7	
Current smoker	15.9	13.5	17.7	15.9	
Unknown	2.3	2.2	2.4	2.2	
Emergency room visit (%)					0.011
No visits	71.7	75.7	69.2	70.6	
1 visit	14.9	13.8	15.2	16.3	
>1 visit	13.4	10.5	15.7	13.1	
Inpatient admissions (%)	15.0	10.0	17.1	18.4	<0.001
Hypoglycemia event (%)	1.8	1.4	1.7	2.7	0.183
Hypoglycemia or outpatient lab (glucose <54 mg/dL) (%)	6.8	4.8	7.8	7.8	0.024
Hyperglycemia event (%)	3.8	2.1	5.1	3.9	0.004
DCSI weighted score (%)					0.458
Score = 0	19.0	18.2	19.7	18.8	
Score = 1	22.4	23.0	21.4	23.7	
Score = 2	17.2	18.9	17.3	14.5	
Score ≥3	41.3	40.0	41.6	42.9	
Frailty index (%)					0.001
Non	19.7	20.4	19.7	18.5	
Pre	32.9	34.9	30.9	34.0	
Mildly	24.3	26.1	24.8	20.6	
Moderately	12.2	11.8	12.2	12.8	
Severe	10.9	6.8	12.4	14.1	
CAN score percentile, probability of death at 1 year (%)					0.047
≤25%	25.9	23.7	27.6	26.1	
26–50%	25.4	26.2	24.8	25.3	
51–75%	24.5	28.2	22.6	22.4	
>75%	24.2	22.0	24.9	26.3	
eGFR (%)					0.333
<45 mL/min/1.73 m^2^	13.9	12.2	14.0	16.5	
45–59 mL/min/1.73 m^2^	15.2	15.5	14.9	15.4	
≥60 mL/min/1.73 m^2^	70.9	72.3	71.2	68.1	
No show rate (%)					<0.001
0%	17.3	23.1	14.6	13.5	
1–20%	65.6	65.6	67.1	62.4	
>20%	17.1	11.3	18.3	24.1	
Medication adherence (%)					0.128
≤50%	21.2	18.1	22.7	23.2	
51–75%	25.7	24.4	25.7	28.0	
76–90%	29.4	32.4	28.8	25.8	
90–100%	23.6	25.1	22.8	23.0	
VA facility complexity level – High (%)	80.2	81.7	79.7	75.9	0.400
Baseline MG (mg/dL)*	175.3 (36.0)	168.6 (31.1)	179.1 (37.9)	178.1 (37.7)	<0.001
Baseline TIR (%)*	58.3 (20.4)	62.3 (19.2)	55.7 (20.5)	57.1 (20.9)	<0.001
Baseline TBR (%)^$^	0.8 [0.2, 2.0]	0.7 [0.2, 1.9]	0.8 [0.2, 2.1]	0.8 [0.2, 2.0]	0.427
Baseline CV*	0.3 (0.1)	0.3 (0.1)	0.3 (0.1)	0.3 (0.1)	0.025

Hypoglycemia and hyperglycemia risk scores were identified using ICD-9/10 diagnostic codes from inpatient or emergency room encounters, recorded in any diagnostic position. Additional identification of hypoglycemia (glucose <54 mg/dL) was based on either diagnostic codes or outpatient laboratory values. Extended care included nursing home consultations or skilled home care consults.

The baseline period for clinical events and laboratory values was defined as one year prior to CGM initiation, except for the Frailty Index and DCSI (24 months) and HbA1c (most recent value before, or within 3 months after initiation). CGM-derived metrics (MG, TIR, TBR, CV) were calculated from the first two weeks of CGM data following initiation.

Insulin pump use was classified separately and excluded from other forms of insulin use. Non-insulin diabetes medication use was defined by the presence of at least one prescription in the year prior to CGM initiation, and included metformin, alpha-glucosidase inhibitors, amylin analogs, DPP-4 inhibitors, meglitinides, sulfonylureas, SGLT2 inhibitors, and thiazolidinediones. The no-show rate reflected the proportion of missed clinic visits out of all scheduled visits during the baseline year. The medication adherence was the mean PDC for statin and anti-hypertensive medications among prevalent users with a prescription during the baseline year.

Continuous variables were reported as means (SD) or medians [interquartile range], and categorical variables as percentages. Group comparisons were performed using ANOVA or Kruskal–Wallis tests for continuous variables, and chi-square or Fisher’s exact tests for categorical variables, as appropriate.

Abbreviations: CAN, care assessment needs; CV, coefficient of variation; DBP, diastolic blood pressure; DCSI, diabetes complication severity index; DM, diabetes mellitus; MG, mean glucose; eGFR, estimated glomerular filtration rate; IQR, inter-quartile range; PCP, primary care provider; LDL-C, low-density lipoprotein cholesterol; SBP, systolic blood pressure; SD, standard deviation; TBR, time-below-range; TIR, time-in-range; VA, Veterans Affairs.

*Mean(SD); ^$^ median [IQR].

### Clustering analysis

Using the cluster stability criterion, we selected *k* = 3 as the optimal number of clusters. This solution minimized both the average raw dissimilarity (mean = 0.13, SD = 0.02) and the normalized dissimilarity (mean = 0.20, SD = 0.03) across 100 bootstrap replicates (S3 Fig). The mean pairwise agreement in cluster assignments was 87%, indicating that individual allocations remained highly consistent under random subsampling (see S1 Appendix, Note 5 for details).

We further evaluated cluster separation by comparing within-cluster and between-cluster distances using the same pairwise distance matrix as in spectral clustering. For *k* = 3, the mean within-cluster distance was 0.175, while the mean between-cluster distance was 0.298, yielding a separation ratio of 1.71, indicating that trajectories within clusters are substantially more similar than those across clusters. Although the separation ratio increased slightly at higher values of *k*, this gain was accompanied by a marked decrease in stability. In contrast, *k* = 3 achieved both high stability and strong separation, supporting its selection as the optimal number of clusters (S5 Table).

**Fig 2:**
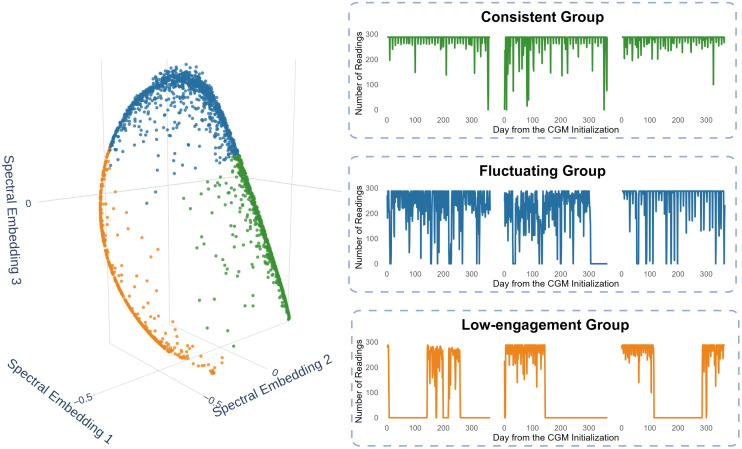
Three-dimensional spectral embeddings of CGM usage trajectories with representative examples for each cluster. Each point represents one individual’s CGM usage trajectory over the first year after device initiation. Colors denote the identified clusters: consistent (green), fluctuating (blue), and low-engagement (orange). Insets show the number of CGM readings per day over time for representative users from each group, illustrating typical usage patterns.

When projected into the three‐dimensional spectral embedding (Fig 2), users formed a convex, disc‐shaped manifold. Most points lay along the periphery, tracing a smooth gradient of CGM engagement. Trajectories sampled around the edge illustrated a clear progression—from robust, near-continuous wear through intermittent use to sparse re-engagement.

We summarized characteristics of the three identified usage clusters in S3 Table and labeled them as *Consistent*, *Fluctuating*, and *Low-engagement* users. Consistent users (*n* = 806) wore their CGM devices nearly continuously over the year (Mean PTC: 93.5%). While some may have brief interruptions in usage, their device engagement during active wear periods was highly consistent, as reflected by near-complete PTC on active days. Fluctuating users (*n* = 1,035) demonstrated intermittent or inconsistent usage. Although their overall coverage remained moderately high (Mean PTC: 78.2%), and the majority would still be classified as adherent users under conventional definitions. Their usage complexity, as captured by RCMSE, was markedly higher than in the other clusters, reflecting greater day-to-day irregularity in CGM engagement. Low-engagement users (*n* = 510) exhibited sparse CGM usage. Their overall CGM coverage was substantially lower (Mean PTC: 33.5%), and their usage complexity was the highest among all clusters. This pattern suggested discontinuation or infrequent re-engagement with the device.

**Fig 3:**
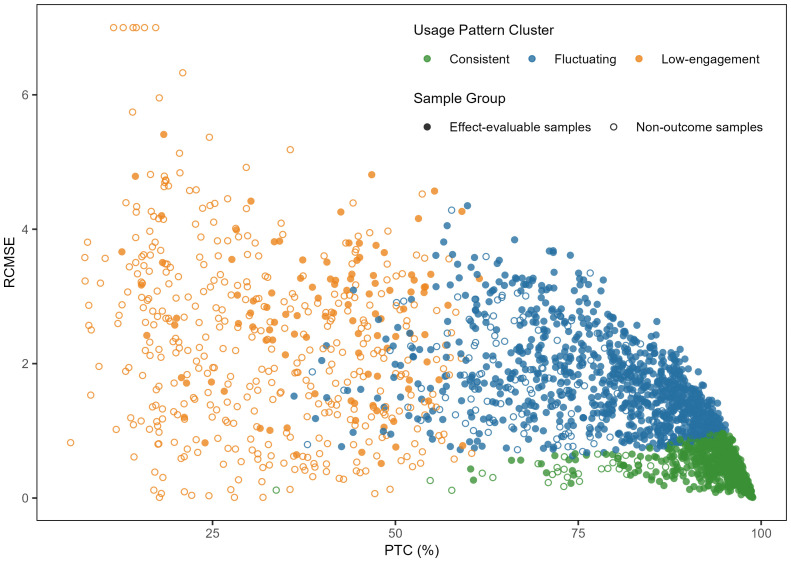
Relationship between PTC and RCMSE, stratified by CGM usage pattern clusters and sample groups. Each point represents an individual’s CGM usage trajectory during the first year after CGM initiation. Points are color-coded by usage pattern clusters—green for consistent, blue for fluctuating, and orange for low-engagement—and are marked by sample group: solid circles indicate individuals with observed glycemic outcomes, and hollow circles indicate samples lack of enough readings during follow-up window. Only individuals with observed outcomes were included in the effect size analysis.

We further visualized PTC against usage complexity (RCMSE) in Fig 3. Even in this two‐dimensional projection, the three clusters remained well separated. PTC and RCMSE showed a negative, nonlinear relationship with a clear funnel pattern: variability in RCMSE is wide at low PTC and tight at high PTC.

### Average potential outcomes across clusters

To adjust for confounding, we included all baseline variables listed in Table 2 (with the exception of index year) as covariates in both the outcome model and the propensity model. The associated nuisance functions, *g*(*D*, *X*) and $m_{j}(X)$ were estimated using four candidate machine-learning algorithms, i.e., elastic net, random forest, support vector machine, and a multilayer perceptron—as described in the Methods. As illustrated in S4 Fig, elastic net and random forest consistently showed the lowest average loss and the smallest variability across resamples, and were therefore chosen for our primary analyses.

We compared the estimated APOs across different combinations of outcome and treatment models, where each was fit using either elastic net or random forest. These comparisons were conducted using 50 repetitions of cross-fitting. Across all outcomes, the estimated APOs were similar across model combinations, with overlapping distributions. Based on these results, the final algorithm selections for each outcome were guided by both stability and performance, and were summarized in S4 Table.

**Fig 4:**
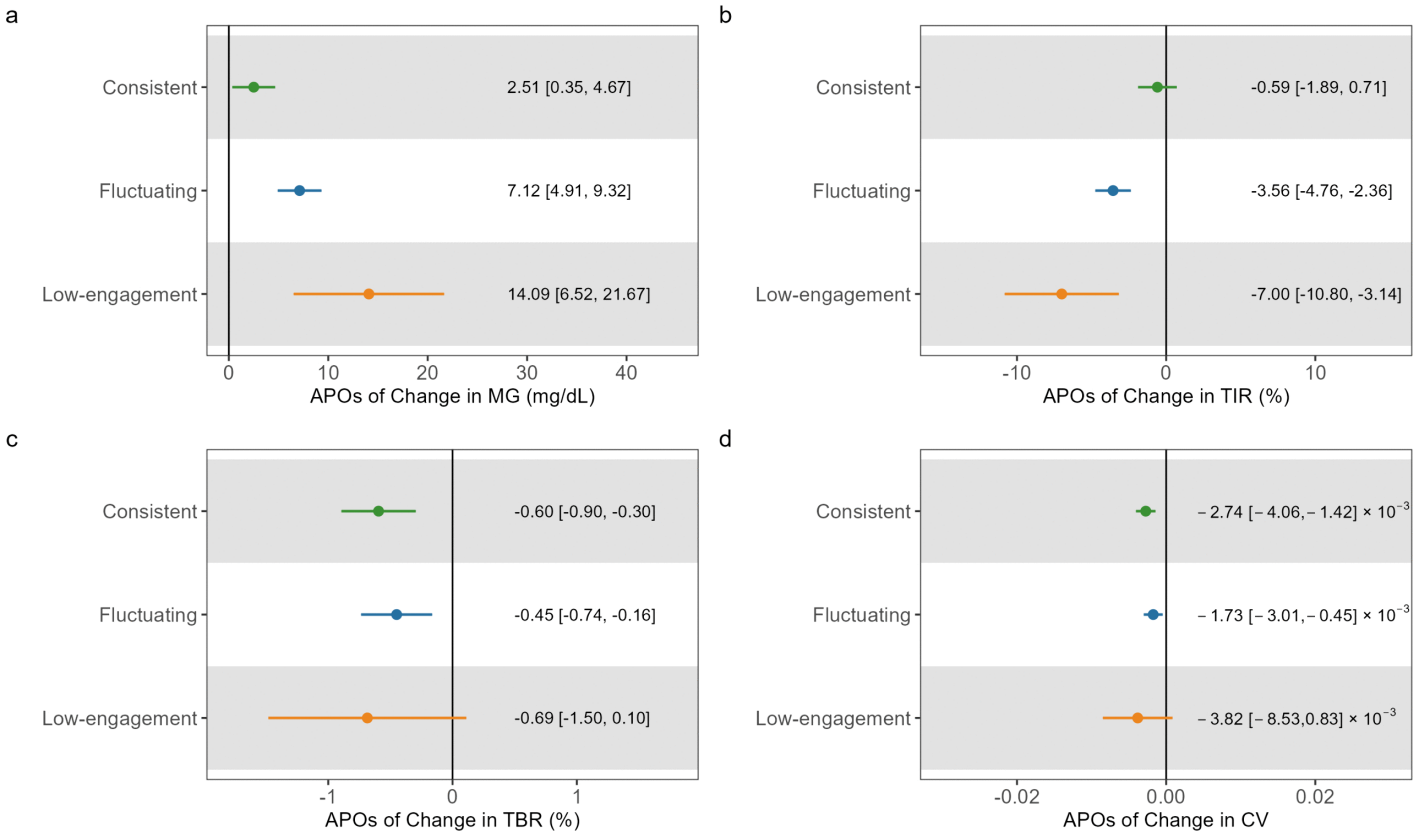
Estimated average potential outcomes (APOs) of glycemic change under different CGM usage patterns. Panels show the estimated APOs for changes in (a) mean glucose (MG), (b) time-in-range (TIR), (c) time-below-range (TBR), and (d) coefficient of variation (CV) over one year of CGM use. Results are stratified by CGM usage patterns identified by spectral clustering (consistent, fluctuating, low-engagement). Point estimates and 95% confidence intervals are displayed. 95% confidence intervals were computed using influence-function-based standard errors, derived from cross-fitted score functions under the assumption of asymptotic normality.

Fig 4 displays the estimated APOs for the change of four glycemic metrics across different CGM usage patterns. For changes in MG and TIR, a clear gradient was observed across the three usage groups identified by spectral clustering, with consistent usage showing the smallest increase in MG (APO = +2.51 mg/dL; 95% CI: 0.35–4.67 mg/dL) and a nonsignificant reduction in TIR (APO = −0.59%, 95% CI: −1.89%–0.71%). Fluctuating usage displayed obvious deterioration in MG (APO = +7.12 mg/dL; 95% CI: 4.91–9.32 mg/dL) and TIR (APO = −3.56%, 95% CI: −4.76%−−2.36%). Low-engagement usage had worse APOs than fluctuating usage, with a substantial increase in MG (APO = +14.09 mg/dL; 95% CI: 6.52–21.67 mg/dL) and a decrease in TIR (APO = −7.00%, 95% CI: −10.80%−−3.14%).

In contrast to conventional adherence cutoffs (PTC or PDC ≥80%), spectral clustering identified a distinct fluctuating subgroup, 73.2% of whom still met the PDC ≥80% criterion, yet experienced significantly greater deteriorations in MG and TIR compared with Consistent users.

### Group average potential outcomes across subgroups

**Fig 5:**
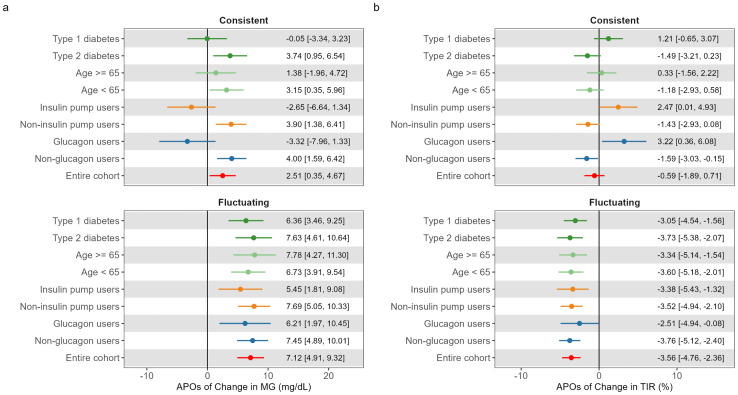
Group average potential outcomes (GAPOs) stratified by clinical subgroups. Panels show the estimated GAPOs of consistent and fluctuating users for changes in (a) mean glucose (MG) and (b) time-in-range (TIR), stratified by diabetes type, age group, insulin pump use, and glucagon use. Point estimates and 95% confidence intervals are displayed. 95% confidence intervals were computed using influence-function-based standard errors, derived from cross-fitted score functions under the assumption of asymptotic normality.

We estimated GAPOs for subgroups defined by diabetes type, age group, insulin pump use, and glucagon use, presenting the results separately for consistent and fluctuating usage patterns (Fig 5). For users belonging to the fluctuating cluster, estimated APOs for change in MG were consistently elevated across subgroups, ranging from 5.45 to 7.78 mg/dL. Similarly, all subgroups were associated with reductions in TIR, with estimates between −3.76% and −2.51%. In contrast, changes in MG and TIR of consistent CGM users varied across subgroups, especially within insulin pump users and glucagon users. The estimated GAPO for change in MG was −2.65 mg/dL among pump users, compared to 3.90 mg/dL among non-pump users. TIR increased by 2.47% in pump users but decreased by 1.43% in non-pump users. A similar pattern was observed for glucagon use. Among glucagon users, the estimated change in MG under consistent usage was −3.32 mg/dL, in contrast to 4.00 mg/dL among non-glucagon users. For TIR, glucagon users showed an estimated improvement of 3.22%, while non-glucagon users experienced a decline of 1.59%.

### Factors associated with CGM usage

To identify baseline characteristics associated with CGM usage patterns, we examined a random forest propensity model trained to predict cluster membership (consistent, fluctuating, low-engagement) using covariates. This model was developed as part of the DML framework and provided insight into which factors differentiate users across the learned clusters. Feature contributions were assessed using SHAP (SHapley Additive exPlanations) values, with a focus on predicting the likelihood of assignment to the consistent usage group [32].

**Fig 6:**
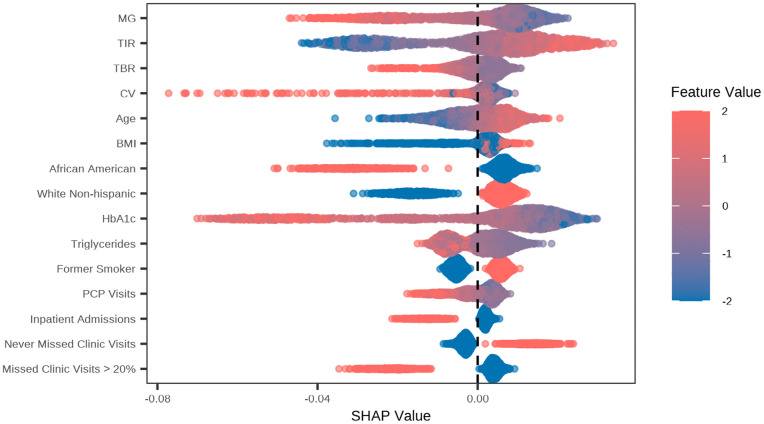
SHAP summary plot for the random forest predicting CGM usage patterns based on baseline covariates. The SHAP values shown here reflect each feature’s contribution to the model’s predicted probability of assignment to the consistent usage class. Only the top 15 features ranked by mean absolute SHAP value are displayed. Feature values are color-coded: continuous variables were standardized and categorical variables were encoded as -2 vs. 2. Positive SHAP values indicate features that increase the likelihood of being assigned to the consistent group, while negative values reduce it. Abbreviations: CV, coefficient of variation; MG, mean glucose; HbA1c, hemoglobin A1c; TBR, time-below-range; PCP, primary care provider.

The SHAP analysis revealed that higher baseline HbA1c and lower TIR were among the strongest predictors of lower probability of consistent CGM use. In addition, greater healthcare utilization, measured by a higher number of primary care visits and inpatient admissions in the prior year, was associated with reduced likelihood of consistent usage. Finally, the number of missed diabetes clinic appointments also negatively contributed to the probability of consistent CGM use (Fig 6).

## Discussion

A major consideration in understanding the effect of CGM on glycemic outcomes is how CGM engagement is defined and characterized over time. Conventional approaches typically quantify adherence using summary measures such as PDC or PTC, often combined with arbitrary thresholds. While these metrics capture overall wear time, they do not fully represent the temporal structure of CGM engagement, including the stability or fluctuation of use across longitudinal follow-up [33]. To address this limitation, we incorporated dissimilarity in use trajectories derived from complexity-adjusted TAOT. Building on prior work demonstrating the importance of complexity correction in time-series distances [34], this approach jointly captures both the amount and temporal variability of CGM use, rather than conditioning on predefined adherence cutoffs. Applying spectral clustering to these trajectory-level representations, we identified three distinct CGM usage phenotypes that differ not only in overall wear time but also in the longitudinal stability of use.

The effectiveness of this approach also reflects the nature of CGM usage data. Unlike physiological signals that often exhibit stable oscillatory structures, CGM usage trajectories are primarily driven by behavioral processes, including adherence, interruptions, and device replacement, which are inherently irregular and often subject-specific. As a result, representations based on frequency decomposition may not provide strong or consistent discriminative structure for distinguishing usage patterns.

Future refinements of CGM usage classification could focus on improving the timeliness and actionability of engagement phenotyping. In particular, identifying engagement patterns earlier in follow-up may help inform earlier identification of individuals at risk of fluctuating or low engagement before these patterns become entrenched. Such early characterization could support targeted education and monitoring strategies aimed at promoting more consistent CGM use in patients most likely to benefit, rather than relying solely on aggregate adherence summaries assessed retrospectively.

The estimated APOs demonstrated a clear, stepwise deterioration of 12-month changes in MG and TIR across CGM usage phenotypes, from consistent to fluctuating to low engagement, indicating that fluctuating and low-engagement phenotypes do not achieve comparable glycemic improvements. By leveraging a DML framework that adjusted for a comprehensive set of baseline CGM-derived and EHR-based covariates, these contrasts are less likely to be driven by measured confounding. The difference in APOs between the consistent and fluctuating phenotypes highlights the critical roles of temporal stability of CGM engagement in maintaining glucose levels over time.

Our findings further demonstrated underlying heterogeneity in the glycemic benefits associated with CGM engagement across patient subgroups. Insulin pump users reflect individuals with more intensive insulin management needs, while those prescribed glucagon are commonly at higher risk for hypoglycemia. In these subgroups, consistent CGM engagement was associated with larger improvements in 12-month glycemic outcomes, indicating that achieving sustained and stable CGM use may be particularly important for patients with greater clnical need. By contrast, low and  fluctuating engagement were not associated with similarly enhanced subgroup-specific benefits. The flutuating engagement group is especially informative because PTC exceeds 78%, yet group-average potential outcomes were comparable to the overall average. This suggests that high PTC alone may not identify patients who are using CGM in a way that translates into meaningful glycemic benefit. Instead, distinguishing sustained from unstable engagement may better identify individuals who may need targeted support to achieve consistent CGM use.

The SHAP analysis indicated factors associated with difficulty in sustaining consistent CGM engagement, with important implications for targeted support. Individuals with poorer glycemic control at initiation were less likely to maintain regular CGM use, which may reflect a combination of greater disease burden, competing self-management demands, or other factors that make sustained engagement more challenging. Greater prior healthcare utilization, including more primary care visits and hospitalization, was also associated with a reduced likelihood of consistent CGM engagement. These measures may reflect broader clinical circumstances and patterns of healthcare interaction that complicate sustained CGM use over time. Missed clinic appointments further highlighted the role of care engagement in shaping CGM use patterns, reinforcing the importance of identifying individuals who may face greater barriers to sustaining long-term CGM engagement.

The goal of this framework is not to provide a fully deployed bedside decision-support tool in its current form, but rather to support population-level characterization, risk stratification, and targeted implementation of CGM adherence support. The observed gradient in glycemic outcomes across Consistent, Fluctuating, and Low-engagement phenotypes suggests that both the amount and stability of CGM use may be clinically meaningful. In addition, baseline factors associated with lower probability of consistent use may help identify patients who are likely to need additional education, troubleshooting, or follow-up after CGM initiation. Before direct clinical deployment, however, the framework should be externally validated and recalibrated in other populations, healthcare systems, and device contexts.

There were two main limitations in this work. First, findings might be biased by unobserved determinants of CGM usage. While the doubly robust property of the DML framework helps mitigate bias from nuisance functions, it is not possible to definitively exclude confounding in observational studies; thus, it is essential to validate the findings through subsequent controlled trials.

Second, many individuals in the low-engagement group were excluded from the effect size analysis due to missing outcome data. Although we applied IPW to account for the missingness, it led to wider confidence intervals in the low-engagement group. Moreover, some individuals might have experienced interruptions in CGM use due to hospitalization or other health-related events, leading to outcome data that were missing not at random. In such cases, IPW could be insufficient to fully correct for the bias, underscoring the need for caution in interpreting the estimated effects for this group. In addition, device model or generation information was unavailable, precluding adjustment for potential differences across Dexcom models. Such differences may introduce heterogeneity in CGM-derived measurements and, potentially, in longitudinal usage behavior.

Our study leveraged a large cohort of U.S. Veterans Affairs patients, who were predominantly older and male. These demographics might not fully represent community or commercially insured populations (e.g., younger adults, females, or non-Veterans). Consequently, the specific adherence phenotypes and their estimated effects on glycemic control might differ in other settings. Future work should validate these trajectory-based clusters in independent cohorts, such as commercially insured CGM users, pediatric populations, and non-VA health systems, to assess the external robustness and potential need for phenotype re-calibration.

## Conclusion

By integrating spectral clustering with DML, we identified three distinct adherence phenotypes and quantified their association with glycemic outcomes. Consistent CGM engagement emerged as the primary driver of glucose stability or improvement over time, while fluctuating and low-engagement patterns were associated with progressive glycemic deterioration. CGM benefits are heterogeneous across patients, and even within the consistent engagement phenotype, greater benefit is observed in specific subgroups, particularly insulin pump users and individuals prescribed glucagon. These findings highlight that the effectiveness of CGM depends not only on access to the device but critically on long-term adherence. Beyond CGM, the analytical framework presented here offers a generalizable approach for characterizing usage phenotypes and linking them to clinical outcomes, with potential applicability to other intermittently used wearable biosensors.

## Supporting information

S1 Fig Illustration of Complexity-adjusted TAOT Distance. (DOCX)

S2 Fig Distribution of CGM coverage metrics during the first year of use across the cohort. (DOCX)

S3 Fig Clustering stability across different numbers of clusters. (DOCX)

S4 Fig Model performance comparisons across prediction tasks using different machine learning algorithms. (DOCX)

S1 Table Chronological overview of selected studies evaluating the effect of CGM. (DOCX)

S2 Table Definitions of CGM-derived metrics used in the analysis. (DOCX)

S3 Table Summary of CGM usage characteristics by cluster. (DOCX)

S4 Table Final machine learning models used in Double Machine Learning estimation of average potential outcomes. (DOCX)

S5 Table Evaluation of clustering performance across different numbers of clusters (*k*). (DOCX)

S1 Appendix Supplementary notes on implementation details. (DOCX)

## References

[1] Beck RW, Riddlesworth T, Ruedy K, Ahmann A, Bergenstal R, Haller S, et al. Effect of Continuous Glucose Monitoring on Glycemic Control in Adults With Type 1 Diabetes Using Insulin Injections: The DIAMOND Randomized Clinical Trial. JAMA. 2017;317(4):371–8. doi: 10.1001/jama.2016.19975 28118453

[2] Lind M, Polonsky W, Hirsch IB, Heise T, Bolinder J, Dahlqvist S, et al. Continuous Glucose Monitoring vs Conventional Therapy for Glycemic Control in Adults With Type 1 Diabetes Treated With Multiple Daily Insulin Injections: The GOLD Randomized Clinical Trial. JAMA. 2017;317(4):379–87. doi: 10.1001/jama.2016.19976 28118454

[3] Reaven PD, Newell M, Rivas S, Zhou X, Norman GJ, Zhou JJ. Initiation of Continuous Glucose Monitoring Is Linked to Improved Glycemic Control and Fewer Clinical Events in Type 1 and Type 2 Diabetes in the Veterans Health Administration. Diabetes Care. 2023;46(4):854–63. doi: 10.2337/dc22-2189 36807492 PMC10260873

[4] Liu TYA, Shpigel J, Khan F, Smith K, Prichett L, Channa R, et al. Use of Diabetes Technologies and Retinopathy in Adults With Type 1 Diabetes. JAMA Netw Open. 2024;7(3):e240728. doi: 10.1001/jamanetworkopen.2024.0728 38446483 PMC10918500

[5] Alsoudi AF, Wai KM, Koo E, Koo E, Mruthyunjaya P, Rahimy E. Reduced rates of diabetic retinopathy complications with use of continuous glucose monitoring. Sci Rep. 2025;15(1):25215. doi: 10.1038/s41598-025-08971-7 40652066 PMC12255799

[6] Majithia AR, Kusiak CM, Armento Lee A, Colangelo FR, Romanelli RJ, Robertson S, et al. Glycemic Outcomes in Adults With Type 2 Diabetes Participating in a Continuous Glucose Monitor-Driven Virtual Diabetes Clinic: Prospective Trial. J Med Internet Res. 2020;22(8):e21778. doi: 10.2196/21778 32856597 PMC7486672

[7] Laffel LM, Kanapka LG, Beck RW, Bergamo K, Clements MA, Criego A, et al. Effect of Continuous Glucose Monitoring on Glycemic Control in Adolescents and Young Adults With Type 1 Diabetes: A Randomized Clinical Trial. JAMA. 2020;323(23):2388–96. doi: 10.1001/jama.2020.6940 32543683 PMC7298603

[8] Martens T, Beck RW, Bailey R, Ruedy KJ, Calhoun P, Peters AL, et al. Effect of Continuous Glucose Monitoring on Glycemic Control in Patients With Type 2 Diabetes Treated With Basal Insulin: A Randomized Clinical Trial. JAMA. 2021;325(22):2262–72. doi: 10.1001/jama.2021.7444 34077499 PMC8173473

[9] Kant R, Antony MA, Geurkink D, Gilreath N, Chandra L, Zipprer E, et al. Real-time continuous glucose monitoring improves glycemic control and reduces hypoglycemia: Real-world data. Prim Care Diabetes. 2022;16(6):786–90. doi: 10.1016/j.pcd.2022.09.005 36117090

[10] Cho SH, Kim S, Lee Y-B, Jin S-M, Hur KY, Kim G, et al. Impact of continuous glucose monitoring on glycemic control and its derived metrics in type 1 diabetes: a longitudinal study. Front Endocrinol (Lausanne). 2023;14:1165471. doi: 10.3389/fendo.2023.1165471 37255973 PMC10225713

[11] Shields S, Thomas R, Durham J, Moran J, Clary J, Ciemins EL. Continuous glucose monitoring among adults with type 2 diabetes receiving noninsulin or basal insulin therapy in primary care. Sci Rep. 2024;14(1):31990. doi: 10.1038/s41598-024-83548-4 39738725 PMC11686249

[12] Lever CS, Williman JA, Boucsein A, Watson A, Sampson RS, Sergel-Stringer OT, et al. Extended use of real-time continuous glucose monitoring in adults with insulin-requiring type 2 diabetes: Results from the first 26 weeks of the 2GO-CGM trial. Diabet Med. 2025;42(5):e70025. doi: 10.1111/dme.70025 40102012 PMC12006558

[13] Panhyar SQ, Memon IH, Qazi A. Continuous glucose monitoring adherence patterns and glycemic variability: CGM adherence and glycemic variability. Journal of Community Health and Medicine. 2025;2(02):136–48.

[14] de Bock M, Cooper M, Retterath A, Nicholas J, Ly T, Jones T, et al. Continuous Glucose Monitoring Adherence: Lessons From a Clinical Trial to Predict Outpatient Behavior. J Diabetes Sci Technol. 2016;10(3):627–32. doi: 10.1177/1932296816633484 26908570 PMC5038543

[15] Murata T, Kuroda A, Matsuhisa M, Toyoda M, Kimura M, Hirota Y, et al. Predictive Factors of the Adherence to Real-Time Continuous Glucose Monitoring Sensors: A Prospective Observational Study (PARCS STUDY). J Diabetes Sci Technol. 2021;15(5):1084–92. doi: 10.1177/1932296820939204 32762345 PMC8442175

[16] Nemlekar PM, Hannah KL, Green CR, Norman GJ. Association between adherence, A1c improvement, and type of continuous glucose monitoring system in people with type 1 diabetes or type 2 diabetes treated with intensive insulin therapy. Diabetes Therapy 15(3), pp. 639–48;2024 doi: 10.1007/s13300-023-01529-8 38289464 PMC10942933

[17] Okuno T, Macwan SA, Miller D, Norman GJ, Reaven P, Zhou JJ. Assessing Patterns of Continuous Glucose Monitoring Use and Metrics of Glycemic Control in Type 1 Diabetes and Type 2 Diabetes Patients in the Veterans Health Care System: Integrating Continuous Glucose Monitoring Device Data with Electronic Health Records Data. Diabetes Technol Ther. 2024;26(11):806–13. doi: 10.1089/dia.2024.0083 38768417 PMC13354035

[18] Okuno T, Sort L, Zhang B, Zhou K, Kitchen M, Li V, et al. Temporal Glycemic Patterns in Type 1 and Type 2 Diabetes: Insights From Extended Continuous Glucose Monitoring. J Diabetes Sci Technol. 2025;:19322968251341264. doi: 10.1177/19322968251341264 40413580 PMC12104196

[19] Keogh EJ, Pazzani MJ. Derivative Dynamic Time Warping. In: Proceedings of the 2001 SIAM International Conference on Data Mining, 2001. 1–11. 10.1137/1.9781611972719.1

[20] Holder C, Middlehurst M, Bagnall A. A review and evaluation of elastic distance functions for time series clustering. Knowl Inf Syst. 2023;66(2):765–809. doi: 10.1007/s10115-023-01952-0

[21] Zhang Z, Tang P, Corpetti T. Time Adaptive Optimal Transport: A Framework of Time Series Similarity Measure. IEEE Access. 2020;8:149764–74. doi: 10.1109/access.2020.3016529

[22] Batista GEAPA, Keogh EJ, Tataw OM, de Souza VMA. CID: An efficient complexity-invariant distance for time series. Data Mining and Knowledge Discovery. 2014;28(3):634–69. doi: 10.1007/s10618-013-0312-3

[23] Wu S-D, Wu C-W, Lin S-G, Lee K-Y, Peng C-K. Analysis of complex time series using refined composite multiscale entropy. Physics Letters A. 2014;378(20):1369–74. doi: 10.1016/j.physleta.2014.03.034

[24] Cai J, Yang Q, Lu J, Shen Y, Wang C, Chen L, et al. Impact of the Complexity of Glucose Time Series on All-Cause Mortality in Patients With Type 2 Diabetes. J Clin Endocrinol Metab. 2023;108(5):1093–100. doi: 10.1210/clinem/dgac692 36458883 PMC10099164

[25] Li C, Ma X, Lu J, Tao R, Yu X, Mo Y, et al. Decreasing complexity of glucose time series derived from continuous glucose monitoring is correlated with deteriorating glucose regulation. Front Med. 2023;17(1):68–74. doi: 10.1007/s11684-022-0955-9 36562949

[26] Ng AY, Jordan MI, Weiss Y. On spectral clustering: Analysis and an algorithm. In: Proceedings of the 15th International Conference on Neural Information Processing Systems: Natural and Synthetic, 2001. 849–56.

[27] von Luxburg U. A tutorial on spectral clustering. Stat Comput. 2007;17(4):395–416. doi: 10.1007/s11222-007-9033-z

[28] Lange T, Roth V, Braun ML, Buhmann JM. Stability-based validation of clustering solutions. Neural Comput. 2004;16(6):1299–323. doi: 10.1162/089976604773717621 15130251

[29] Chernozhukov V, Chetverikov D, Demirer M, Duflo E, Hansen C, Newey W, et al. Double/debiased machine learning for treatment and structural parameters. The Econometrics Journal. 2018;21(1):C1–68. doi: 10.1111/ectj.12097

[30] Bach P, Chernozhukov V, Kurz MS, Spindler M. DoubleML – an object-oriented implementation of double machine learning in Python. Journal of Machine Learning Research. 2022;23(53):1–6.

[31] Rosenbaum PR, Rubin DB. The central role of the propensity score in observational studies for causal effects. Biometrika. 1983;70(1):41–55. doi: 10.1093/biomet/70.1.41

[32] Lundberg SM, Lee S. A unified approach to interpreting model predictions. In: arXiv preprint, 2017. https://doi.org/arXiv:1705.07874

[33] Hirsch IB, Garg SK, Repetto E, Snell-Bergeon J, Ulmer B, Perkins C, et al. Continuous Glucose Monitoring Frequency and Glycemic Control in People With Type 2 Diabetes. JAMA Netw Open. 2025;8(10):e2539278. doi: 10.1001/jamanetworkopen.2025.39278 41171276 PMC12579344

[34] Hall H, Perelman D, Breschi A, Limcaoco P, Kellogg R, McLaughlin T, et al. Glucotypes reveal new patterns of glucose dysregulation. PLoS Biol. 2018;16(7):e2005143. doi: 10.1371/journal.pbio.2005143 30040822 PMC6057684

