# Supplementary material for: Digital phenotyping of CGM engagement reveals distinct glycemic outcomes
Source: PLOS Digit Health. 2026 Jul 23;5(7):e0001505. doi: 10.1371/journal.pdig.0001505 (PMC13395450; doi:10.1371/journal.pdig.0001505)
Supplement: S1 Fig — (DOCX) [file pdig.0001505.s001.docx]

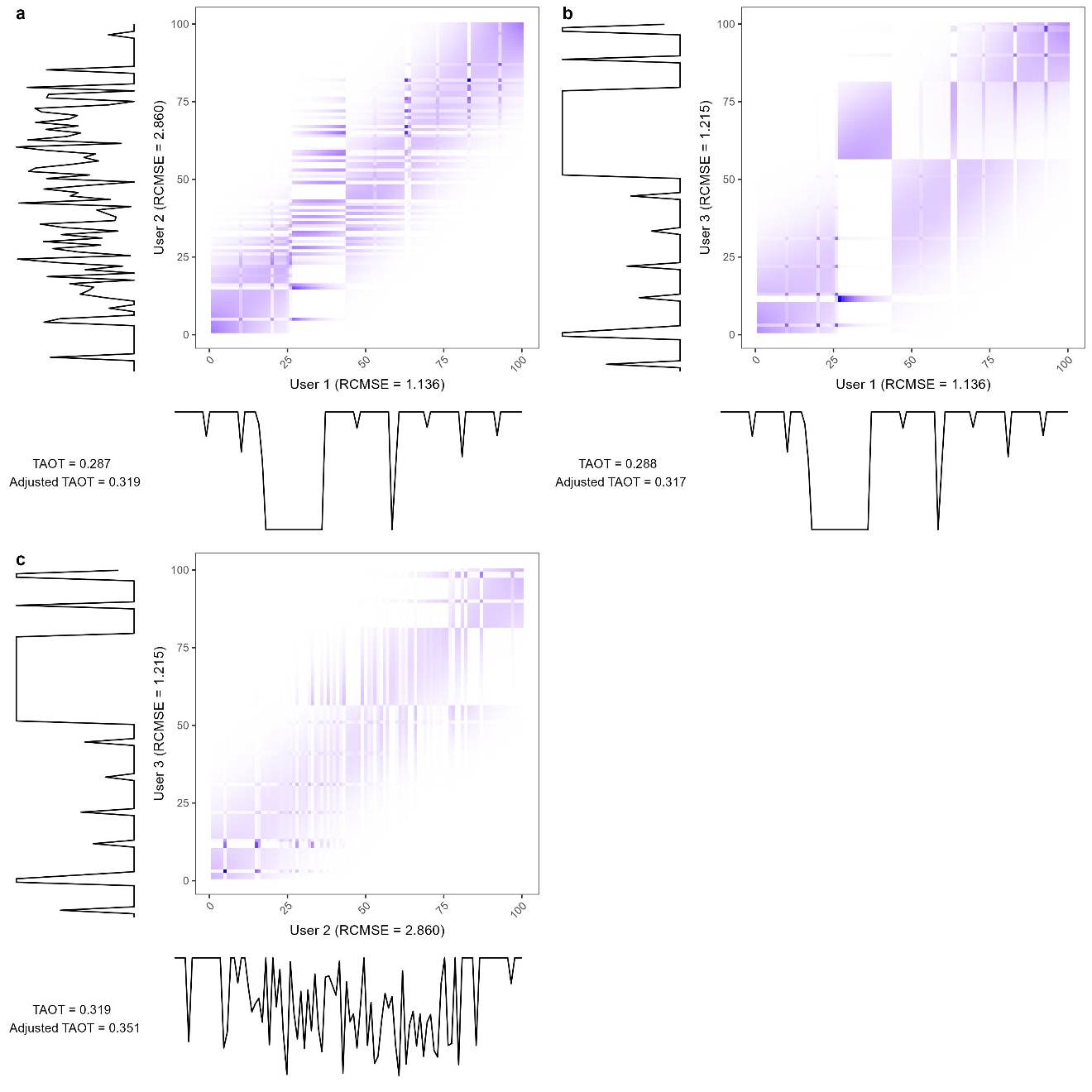


**S1 Fig. Illustration of Complexity-adjusted TAOT Distance.** This figure compares the TAOT distance with and without complexity adjustment across three users' CGM usage trajectories over a 100-day period. Each subplot (a–c) shows a pairwise comparison between two users: the heat plot represents the pairwise TAOT alignment, flanked by usage trajectories and annotated with each user's RCMSE. TAOT captures local discrepancies in wear duration through many-to-many alignment, while RCMSE quantifies the structural irregularity or complexity of each trajectory. Before adjustment, Users 1 and 2 appear closer in unadjusted TAOT due to similar durations of device wear during corresponding time windows. However, User 2 exhibits substantially more irregular use behavior than both Users 1 and 3. After complexity adjustment, Users 1 and 3 are recognized as more similar due to their simple and regular usage patterns.
