# Supplementary material for: Digital phenotyping of CGM engagement reveals distinct glycemic outcomes
Source: PLOS Digit Health. 2026 Jul 23;5(7):e0001505. doi: 10.1371/journal.pdig.0001505 (PMC13395450; doi:10.1371/journal.pdig.0001505)
Supplement: S2 Fig — (DOCX) [file pdig.0001505.s002.docx]

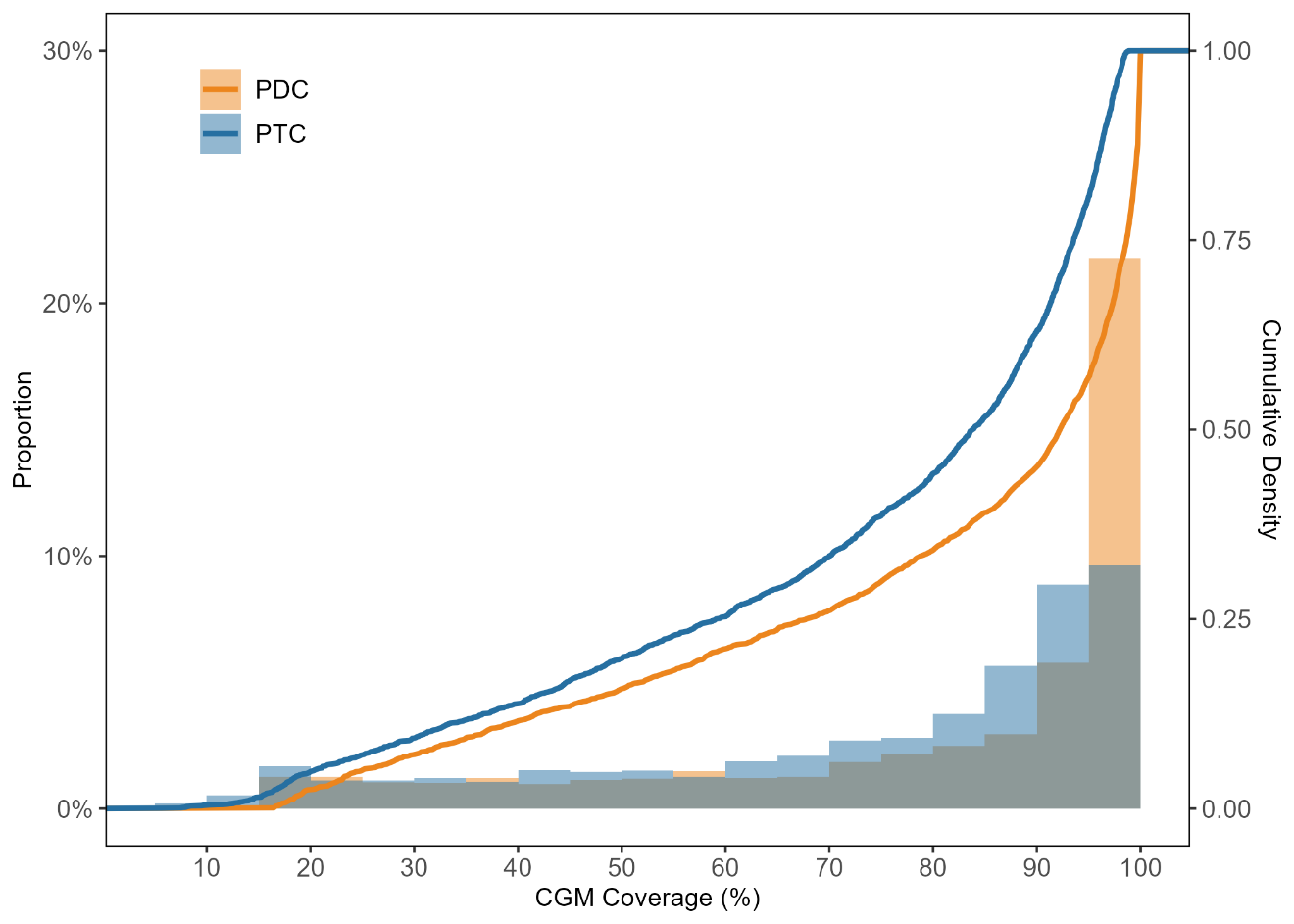


**S2 Fig. Distribution of CGM coverage metrics during the first year of use across the cohort.** Histograms and cumulative density curves represent the proportion of day covered (PDC, colored in orange) and the proportion of time covered (PTC, colored in blue) over the first year following CGM initiation.
