# Supplementary material for: Digital phenotyping of CGM engagement reveals distinct glycemic outcomes
Source: PLOS Digit Health. 2026 Jul 23;5(7):e0001505. doi: 10.1371/journal.pdig.0001505 (PMC13395450; doi:10.1371/journal.pdig.0001505)
Supplement: S3 Fig — (DOCX) [file pdig.0001505.s003.docx]

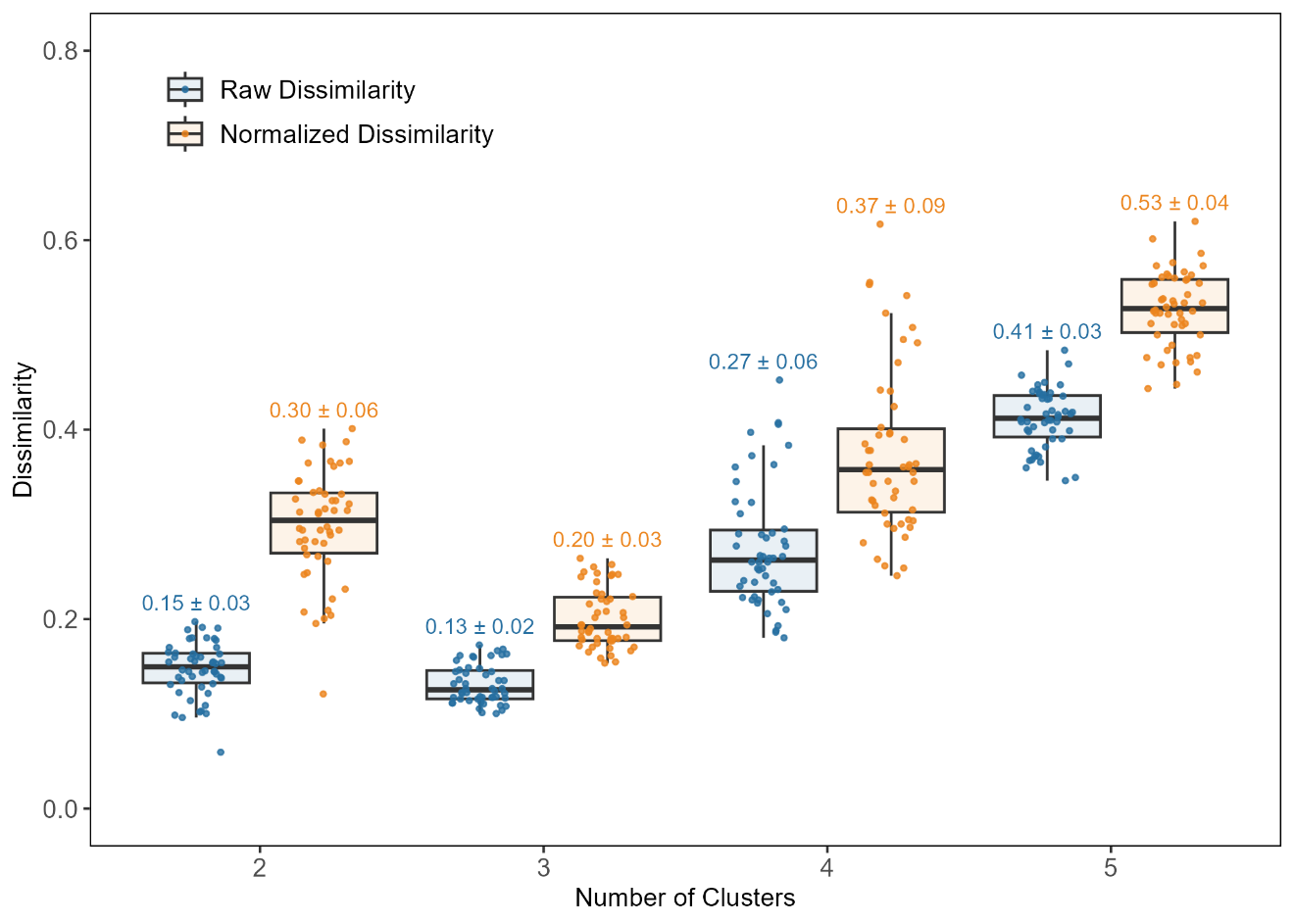


**S3 Fig. Clustering stability across different numbers of clusters.** Raw and normalized dissimilarity metrics were used to assess the stability of spectral clustering solutions across varying numbers of clusters ($k=2$ to $5$). For each $k$, clustering was repeated across 50 random train-test splits. Raw dissimilarity (colored in blue) measures the absolute disagreement between predicted and re-estimated cluster labels in the test set, while normalized dissimilarity (color in orange) accounts for chance agreement.
