# Supplementary material for: Digital phenotyping of CGM engagement reveals distinct glycemic outcomes
Source: PLOS Digit Health. 2026 Jul 23;5(7):e0001505. doi: 10.1371/journal.pdig.0001505 (PMC13395450; doi:10.1371/journal.pdig.0001505)
Supplement: S4 Fig — (DOCX) [file pdig.0001505.s004.docx]

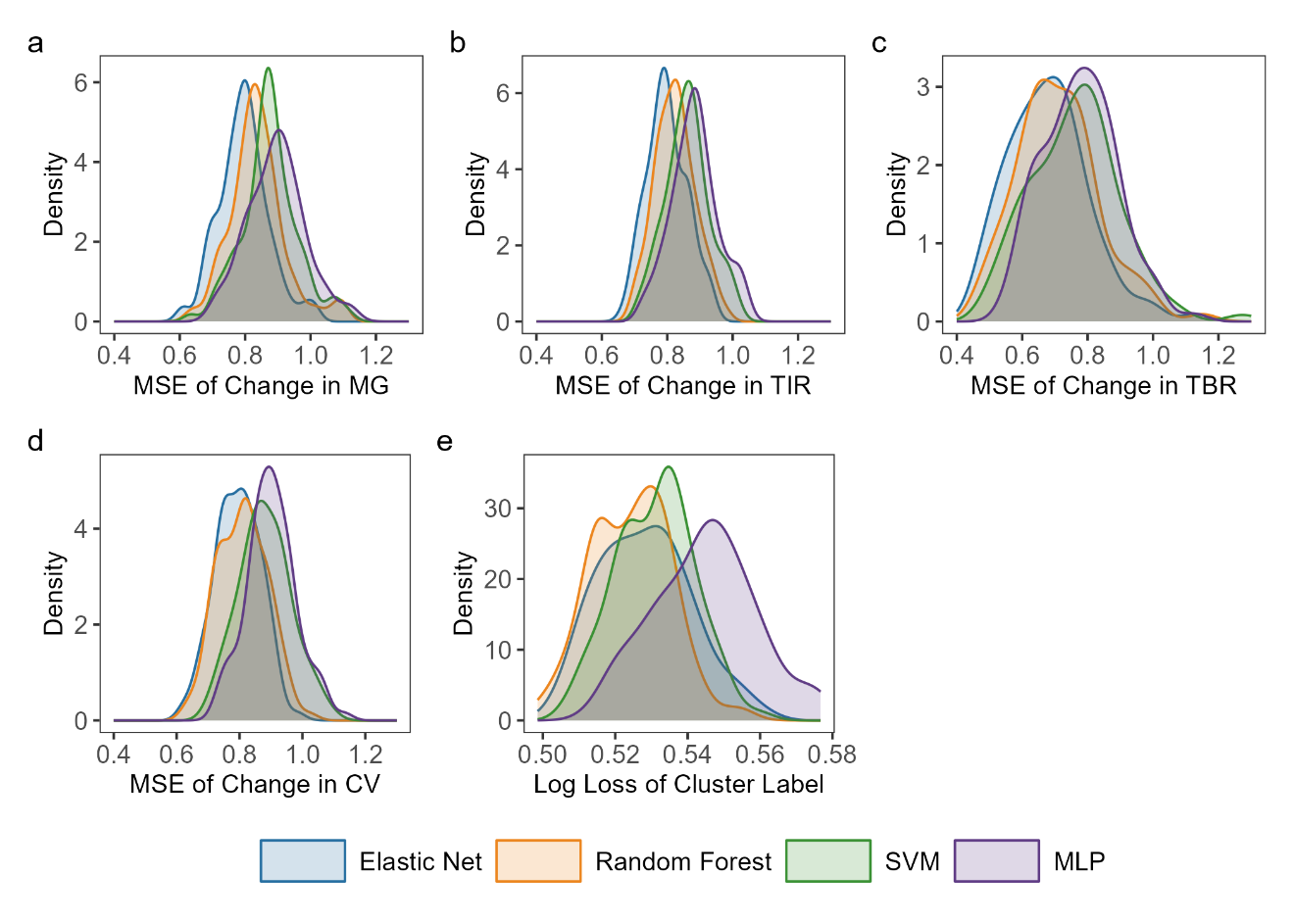


**S4 Fig. Model performance comparison across prediction tasks using different machine learning algorithms.** Panels (a)–(d) show the distributions of mean squared error (MSE) for predicting changes in CGM-derived metrics using covariates and CGM usage patterns as predictors: Change in (a) mean glucose, (b) time-in-range, (c) time-below-range, and (d) coefficient of variation. Panel (e) presents the distribution of log loss for a classification task, predicting CGM usage cluster with covariates. Performance distributions were obtained from 20 repetitions of 5-fold cross-validation, and are shown across train-test splits for four models: Elastic Net (blue), Random Forest (orange), Support Vector Machine (green), and Multi-layer Perceptron (purple). Shaded areas represent kernel density estimates over all repetitions.
