# Supplementary material for: Digital phenotyping of CGM engagement reveals distinct glycemic outcomes
Source: PLOS Digit Health. 2026 Jul 23;5(7):e0001505. doi: 10.1371/journal.pdig.0001505 (PMC13395450; doi:10.1371/journal.pdig.0001505)
Supplement: S1 Table — (DOCX) [file pdig.0001505.s005.docx]

S1 Table Chronological overview of selected studies evaluating the effect of CGM

| **Study** | **Design** | **Population** | **n** | **Duration** | **Device** | **Usage** | **CGM group outcome** |
| --- | --- | --- | --- | --- | --- | --- | --- |
| Lind et al. (2017) | RCT | T1D, MDI | 161 | 26 weeks | Dexcom G4 | Mean PTC = 87.8% (SD 13.4%) | HbA1c ↓ 0.68% |
| Beck et al. (2017) | RCT | T1D, MDI | 158 | 24 weeks | Dexcom G4 | Median PTC = 96% (IQR 90–98%) | HbA1c ↓ 1.0%; TIR ↑ 5.3% |
| Majithia et al. (2020) | Single-arm trial | T2D | 55 | 4 months | Dexcom G6 | Mean PTC = 94.8% (SD 8.2%) | HbA1c ↓ 1.6%; TIR ↑ 10.2% |
| Laffel et al. (2020) | RCT | T1D | 153 | 26 weeks | Dexcom G5 | Median PDC = 90% (IQR 61–100%) | HbA1c ↓ 0.4%; TIR ↑ 6% |
| Martens et al. (2021) | RCT | T2D on basal insulin | 175 | 8 months | Dexcom G6 | Median PDC = 87% (IQR 73–94%) | HbA1c ↓ 1.1%; TIR ↑ 19% |
| Kant et al. (2022) | Observational | T1D + T2D on MDI/CSII | 91 | ≥ 3 months | Dexcom G5/G6 | PTC ≥ 75% | HbA1c ↓ 0.42%; TIR ↑ 0.6% |
| Cho et al. (2023) | Observational | T1D | 465 | 12 months | Multiple | Mean PTC = 87.5% (SD 14.2%) | HbA1c ↓ 0.12%; TIR ↑ 2.9% |
| Shields et al. (2024) | Observational | T2D on non-/basal insulin | 182 | 3 months | Dexcom G6 | PTC ≥ 70% | HbA1c ↓ 1.3%; TIR ↑ 22.2% |
| Lever et al. (2025) | RCT | T2D on non-/basal insulin | 67 | 26 weeks | Dexcom G6 | Median PTC ≥ 90% | HbA1c ↓ 1.1%; TIR ↑ 15% |

Only the first author is listed for brevity; full author names can be found in the references. Values such as “HbA1c↓1.0%” denote within-group improvement from baseline.

Abbreviations: CSII, continuous subcutaneous insulin infusion; HbA1c, hemoglobin A1c; IIT, intensive insulin therapy; MDI, multiple daily insulin injections; PDC, proportion of days covered; PTC, proportion of time covered; TIR, time-in-range.
