# Supplementary material for: Digital phenotyping of CGM engagement reveals distinct glycemic outcomes
Source: PLOS Digit Health. 2026 Jul 23;5(7):e0001505. doi: 10.1371/journal.pdig.0001505 (PMC13395450; doi:10.1371/journal.pdig.0001505)
Supplement: S2 Table — (DOCX) [file pdig.0001505.s006.docx]

S2 Table Definitions of CGM-derived metrics used in the analysis.

| **Term** | **Definition** |
| --- | --- |
| Mean glucose (MG) | The arithmetic mean of all CGM readings recorded. |
| Time-in-range (TIR) | The percentage of time during which CGM glucose values fall within the target range of 70–180 mg/dL (3.9–10.0 mmol/L). |
| Time-below-range (TBR) | The percentage of time during which CGM glucose values are below 70 mg/dL (3.9 mmol/L). |
| Coefficient of variation (CV) | The ratio of the standard deviation to the mean of daily CGM glucose readings, representing glycemic variability. |
| Proportion of days covered (PDC) | The proportion of days within the observation window on which at least one CGM reading was recorded. |
| Proportion of time covered (PTC) | The proportion of the total observation period during which glucose measurements were available, calculated as the number of observed readings divided by the maximum possible number of readings. |
| PTC on active days | The proportion of time covered by CGM readings during days with at least one CGM reading, calculated as the total number of readings on those days divided by the maximum possible number of readings during those days. |
