# Supplementary material for: Digital phenotyping of CGM engagement reveals distinct glycemic outcomes
Source: PLOS Digit Health. 2026 Jul 23;5(7):e0001505. doi: 10.1371/journal.pdig.0001505 (PMC13395450; doi:10.1371/journal.pdig.0001505)
Supplement: S3 Table — (DOCX) [file pdig.0001505.s007.docx]

S3 Table Summary of CGM usage characteristics by cluster

| **Variable** | **Cluster 1 (n = 806)** | **Cluster 2 (n = 1,035)** | **Cluster 3 (n = 510)** |
| --- | --- | --- | --- |
| Cluster Name | Consistent | Fluctuating | Low-engagement |
| PTC (%) | 93.5 ± 6.3 | 78.2 ± 12.0 | 33.5 ± 13.5 |
| PDC (%) | 97.1 ± 6.1 | 86.7 ± 12.2 | 40.1 ± 15.9 |
| RCMSE | 0.46 ± 0.23 | 1.70 ± 0.67 | 2.38 ± 1.33 |
| PTC on Active Days (%) | 96.4 ± 2.3 | 90.2 ± 5.6 | 83.5 ± 12.4 |

Values are mean$\pm$SD.
