# Supplementary material for: Digital phenotyping of CGM engagement reveals distinct glycemic outcomes
Source: PLOS Digit Health. 2026 Jul 23;5(7):e0001505. doi: 10.1371/journal.pdig.0001505 (PMC13395450; doi:10.1371/journal.pdig.0001505)
Supplement: S4 Table — (DOCX) [file pdig.0001505.s008.docx]

S4 Table Final machine learning models used in Double Machine Learning estimation of average potential outcomes.

| **Outcome** | **Propensity model** $\boldsymbol{m}_{\boldsymbol{j}}\left( \boldsymbol{X} \right)$ | **Outcome model** $\boldsymbol{g}\left( \boldsymbol{D,X} \right)$ |
| --- | --- | --- |
| Change in MG | RF (n_estimator = 500, max_depth = 5) | RF (n_estimator = 800, max_depth = 10) |
| Change in TIR | RF (n_estimator = 500, max_depth = 5) | RF (n_estimator = 200, max_depth = 3) |
| Change in TBR | Enet (C = 0.01, L1 ratio = 0) | RF (n_estimator = 1000, max_depth = 5) |
| Change in CV | Enet (C = 0.01, L1 ratio = 0) | RF (n_estimator = 800, max_depth = 10) |

Model hyperparameters were selected based on cross-validation. $m_{j}\left( X \right)$ denotes the classification model used to predict usage pattern assignment; $g\left( D,X \right)$ denotes the regression model used to estimate APOs.

Abbreviations: RF, Random Forest; Enet, Elastic Net; n_estimator, number of trees in the random forest; max_depth, maximum tree depth; C, inverse regularization strength.
