# Supplementary material for: Digital phenotyping of CGM engagement reveals distinct glycemic outcomes
Source: PLOS Digit Health. 2026 Jul 23;5(7):e0001505. doi: 10.1371/journal.pdig.0001505 (PMC13395450; doi:10.1371/journal.pdig.0001505)
Supplement: S5 Table — (DOCX) [file pdig.0001505.s009.docx]

S5 Table Evaluation of clustering performance across different numbers of clusters (k).

| **# of Clusters** | **Within-cluster dist.**  **(Mean)** | **Between-cluster dist.**  **(Mean)** | **Separation ratio** | **Stability**  **(Mean** $\boldsymbol{\pm}$ **SD)** |
| --- | --- | --- | --- | --- |
| **2** | 0.193 | 0.299 | 1.549 | 0.85$\pm$ 0.03 |
| **3** | 0.175 | 0.298 | 1.706 | 0.87$\pm$ 0.02 |
| **4** | 0.175 | 0.287 | 1.644 | 0.73$\pm$ 0.06 |
| **5** | 0.165 | 0.289 | 1.752 | 0.59$\pm$ 0.03 |

Mean within-cluster and between-cluster distances were computed using the same pairwise distance matrix as in spectral clustering. The separation ratio is defined as the ratio of mean between-cluster distance to mean within-cluster distance, with higher values indicating better separation. Stability is reported as the mean ± standard deviation of agreement across 50 random train–test splits, where agreement is defined as the proportion of matched cluster assignments between projected and independently estimated test-set labels.
