# Supplementary material for: Digital phenotyping of CGM engagement reveals distinct glycemic outcomes
Source: PLOS Digit Health. 2026 Jul 23;5(7):e0001505. doi: 10.1371/journal.pdig.0001505 (PMC13395450; doi:10.1371/journal.pdig.0001505)
Supplement: S1 Appendix — (DOCX) [file pdig.0001505.s010.docx]

**Supplementary Notes for “Digital Phenotyping Of CGM Engagement Reveals Distinct Glycemic Outcomes”**

Table of Contents

[Supplementary Notes 2](#_Toc228027621)

[Note 1. Calculation of Time Adaptive Optimal Transport distance 2](#_Toc228027622)

[Note 2. Calculation of Refined Composite Multiscale Entropy 4](#_Toc228027623)

[Note 3. Spectral clustering implementation 6](#_Toc228027624)

[Note 4. Hyperparameter search grid 7](#_Toc228027625)

[Note 5. Stability-based assessment of the number of clusters 9](#_Toc228027626)

# **Supplementary Notes**

## **Note 1. Calculation of Time Adaptive Optimal Transport distance**

Time Adaptive Optimal Transport (TAOT) measures the similarity between time series by considering both observed values and their temporal locations jointly. For each time series of length $T$, we denote the observed values as $a=\left( a_{1},\ldots,a_{T} \right)$ and the corresponding time coordinates as $t=\left( t_{1},\ldots,t_{T} \right)$. Each observation $\left( a_{i},t_{i} \right)$ is treated as a realization of a random variable, with an associated probability mass $p_{i}$. That is, the joint distribution over value-time pairs is defined as:

$$P\{\left( a,t \right)=\left( a_{i},t_{i} \right)\}=p_{i},\quad\text{for }i=1,\ldots,T.$$

Given two such time series $A=\left( a,t \right)$ and $B=\left( b,t \right)$ both of length $T$, TAOT seeks an optimal transport plan that aligns the distributions while respecting both value and temporal differences. This is achieved by solving an optimal transport problem with a cost function that incorporates both signal and time differences:

$$M\left( i,j \right)=\left( a_{i}-b_{j} \right)^{2}+w\cdot\left( t_{i}-t_{j} \right)^{2},$$

where $w\geq0$ controls the importance of temporal alignment.

Then the optimal transport distance under this cost matrix $M\left( i,j \right)$ is defined as:

$$d_{M}\left( A,B \right)=\min_{P} \sum_{i,j=1}^{T} p_{ij}m_{ij}$$

where $p_{ij}$denotes the amount of probability mass transported from $\left( a_{i},t_{i} \right)$ to $\left( b_{j},t_{j} \right)$, and $P$ is the set of all joint distributions with marginals $p_{a}$ and $p_{b}$. That is,

$$\sum_{j=1}^{d} p_{ij}=p_{a_{i}},\quad\sum_{i=1}^{d} p_{ij}=p_{b_{j}},\quad\text{with }p_{ij}\geq0.$$

Considering efficiency, the TAOT distance was calculated by the Sinkhorn algorithm, where the regularization coefficient was set as 5, and the tolerance was set as 0.01. We set the weight $w=23.04$ such that two non-wear observations (i.e., values of zero) were matched only if they were no more than 60 days apart. This enforced a temporal locality constraint, preventing implausible alignments between observations that are too distant in time.

## **Note 2. Calculation of Refined Composite Multiscale Entropy**

Refined composite multiscale entropy (RCMSE) is a popular method for characterizing the complexity of time series. Let $x=\{x_{1},x_{2},\ldots,x_{T}\}$ represent a time series of length $T$. To calculate the RCMSE of this time series, it is necessary to specify the scale factor $\tau$, the length of template vectors $m$, and the tolerance $r$.

We first calculate the coarse-grained time series. The $k$-th coarse-grained time series is

$$y_{k}^{\left( \tau\right)}=\{y_{k,1}^{\left( \tau\right)},y_{k,2}^{\left( \tau\right)},\cdots,y_{k,p}^{\left( \tau\right)}\}$$

where $y_{k,j}^{\left( \tau\right)}=\frac{1}{\tau}\sum_{i=\left( j-1 \right)\tau+k}^{j\tau+k-1} x_{i}$ ($1\leq j\leq\frac{T}{\tau},1\leq k\leq\tau$).

For each coarse-grained time series, the number of matched pairs is calculated. Take the $k$-th coarse-grained time series $y_{k}^{\left( \tau\right)}$ as an example. We construct $p-m$ template vectors

$$y_{kl}^{\left( \tau,m \right)}=\{y_{k,l}^{\tau},y_{k,l+1}^{\tau},\ldots,y_{k,l+m-1}^{\tau}\},\quad1\leq l\leq p-m.$$

A match occurs when the $L^{\infty}$norm distance between $\left( y_{kl}^{\left( \tau,m \right)},y_{kl^{'}}^{\left( \tau,m \right)} \right)$ is smaller than a predefined tolerance $r$. Let $n_{k,\tau}^{m}$ represent the total number of $m$-dimensional matched vector pairs.

At a scale factor of $\tau$, $n_{k,\tau}^{m+1}$ and $n_{k,\tau}^{m}$ are calculated for all $\tau$ coarse-grained series. Then the RCMSE is defined as:

$$\text{RCMSE}\left( x,\tau,m,r \right)=-\ln\left( \frac{\sum_{k=1}^{\tau} n_{k,\tau}^{m+1}}{\sum_{k=1}^{\tau} n_{k,\tau}^{m}} \right).$$

In RCMSE, $\tau$ controls the scale of coarse-graining, allowing the evaluation of the time series' complexity across different temporal scales. In the context of CGM usage, we considered scale factors $\tau=7$ and $\tau=10$ to capture weekly and 10-day sensor replacement cycles, respectively. The final complexity measure was computed as the sum of RCMSE values across these two scales. The parameter $m=1$ was selected based on the length of the time series, following the guideline that the series length should fall within the range of ${10}^{m}$ to ${30}^{m}$. The tolerance parameter $r=15$ was chosen to minimize the occurrence of undefined entropy.

In practice, we observed that patients with prolonged periods of non-wear exhibited extremely low usage complexity, which did not reflect meaningful usage patterns. To mitigate the impact of extended non-wear on the RCMSE calculation, we excluded segments with more than seven consecutive days of non-wear from the analysis. This step does not compromise the validity of the results, as RCMSE is independent of data length when the time series is sufficiently characterized.

## **Note 3. Spectral clustering implementation**

Spectral clustering is a simple yet effective approach to identify clusters in data. In our study, we applied spectral clustering using a distance matrix derived from a combination of TAOT and RCMSE measures. A fully connected graph was constructed, where the similarity of each pair of usage trajectories was calculated using the Gaussian similarity function:

$$s\left( x_{i},x_{j} \right)=\exp\left( -\frac{\left. |d\left( x_{i},x_{j} \right) \right.|^{2}}{2\sigma^{2}} \right),\quad i,j=1,2,\ldots,N,$$

where $d\left( x_{i},x_{j} \right)$ denotes the distance between trajectories $x_{i}$ and $x_{j}$. The scaling parameter $\sigma$ was defined as the average of the distances from $\boldsymbol{x}_{\boldsymbol{i}}$ and $\boldsymbol{x}_{\boldsymbol{j}}$ to their respective $\sqrt{N}$ nearest neighbors. During prediction, $\sigma$ for each test sample was defined as the average distance to its $\sqrt{N}$ nearest training samples. We then performed normalized spectral clustering, where cluster assignments were obtained by applying k-means to the spectral embeddings using 10 random initializations.

## **Note 4. Hyperparameter search grid**

For each machine learning algorithm, we performed hyperparameter tuning using grid search with 5-fold cross-validation. The search space for each model class is shown below in Python-style pseudocode for reproducibility.

1. Outcome Models

param_grid = {

"enet": [

{"c": c, "l1_ratio": l1}

for c in [0.001, 0.01, 0.1, 1]

for l1 in [0.0, 0.3, 0.5, 0.7, 1.0]

],

"rf": [

{"n_estimators": n, "max_depth": d}

for n in [10, 100, 200, 500]

for d in [3, 5, 7]

],

"svm": [

{"C": c}

for c in [0.1, 1, 3]

],

"mlp": [

{

"hidden_layer_sizes": h,

"alpha": a,

"learning_rate_init": r,

"activation": f,

"learning_rate": lr

}

for h in [(32,), (64,), (16, 16), (32, 32)]

for a in [0.0001, 0.0005, 0.001]

for r in [0.00001, 0.0001, 0.001]

for f in ["tanh"]

for lr in ["adaptive"]

]

}

1. Propensity Score Models

param_grid = {

"enet": [

{"alpha": c, "l1_ratio": l1}

for c in [0.001, 0.01, 0.1, 1]

for l1 in [0.0, 0.3, 0.5, 0.7, 1.0]

],

"rf": [

{"n_estimators": n, "max_depth": d}

for n in [200, 500, 800, 1000]

for d in [3, 5, 10, 15]

],

"svm": [

{"C": c}

for c in [0.05, 0.1, 1]

],

"mlp": [

{

"hidden_layer_sizes": h,

"alpha": a,

"learning_rate_init": r,

"activation": f,

"learning_rate": lr

}

for h in [(32,), (64,), (16, 16), (32, 32)]

for a in [0.0005, 0.001, 0.005, 0.01]

for r in [0.00001, 0.0001, 0.001]

for f in ["tanh"]

for lr in ["adaptive"]

]

}

## **Note 5. Stability-based assessment of the number of clusters**

We used a stability-based validation approach to assess the reproducibility of clustering solutions across candidate numbers of clusters. Following Lange et al., cluster stability was defined based on the reproducibility of clustering assignments on an independent sample, where the preferred number of clusters is the solution with the lowest expected disagreement across repeated data splits. This framework treats instability as a classification risk between two independently obtained cluster labelings, after optimally aligning cluster labels to account for arbitrary label permutations.

For each candidate number of clusters $k$, we repeatedly divided the data into two non-overlapping halves. Spectral clustering was first performed on the training set using the pairwise distance matrix to obtain cluster assignments. To assign cluster labels to individuals in the test set, we computed the pairwise distances between test and training samples and used these distances to project test samples onto the training-derived clustering structure, assigning each test sample to the most similar cluster based on its relationship to the training data.

Independently, spectral clustering was also performed directly on the test set using its own distance matrix. We then compared the predicted test-set labels with the independently estimated test-set labels.

Because cluster labels are arbitrary up to permutation, label agreement was computed after optimal label matching. Specifically, we constructed a confusion matrix between the two test-set labelings and used the Hungarian algorithm to find the permutation that maximized overlap. Stability was then defined as the proportion of matched labels after this optimal alignment. Equivalently, dissimilarity was defined as $1-$stability, corresponding to the proportion of mismatched labels.

Because the raw dissimilarity is not directly comparable across different values of $k$, we further normalized it against the dissimilarity expected under random cluster assignment. Specifically, when $k$ is small, even random labelings may show non-negligible agreement after optimal label matching, which constrains the range of possible dissimilarity values. Therefore, for each candidate $k$, we generated 1,000 random cluster assignments and computed their average dissimilarity after optimal label alignment. The resulting baseline dissimilarities were 0.51, 0.35, 0.27, and 0.22 for $K=2,3,4,$and $5$, respectively. The normalized dissimilarity was then calculated as the raw dissimilarity divided by the corresponding random-labeling baseline for that value of $K$. This normalization places dissimilarity values for different $k$ on a more comparable scale.

For $k=3$, the mean agreement across repeated splits was 0.87, corresponding to a mean disagreement of 0.13. This indicates that 87% of test-set individuals received the same cluster assignment when comparing labels projected from the training-derived solution with labels obtained by independently clustering the test set. Thus, individual allocations were highly reproducible under resampling. After normalization, $k=3$remained the solution with the lowest dissimilarity, indicating that the selection is robust to scale differences across candidate values of $k$.

Although $k=5$ showed a slightly higher separation ratio, its stability dropped substantially, suggesting over-fragmentation rather than reproducible structure.
